# Bayesian functional regression as an alternative statistical analysis of high-throughput phenotyping data of modern agriculture

**DOI:** 10.1186/s13007-018-0314-7

**Published:** 2018-06-11

**Authors:** Abelardo Montesinos-López, Osval A. Montesinos-López, Gustavo de los Campos, José Crossa, Juan Burgueño, Francisco Javier Luna-Vazquez

**Affiliations:** 1 Departamento de Matemáticas, Centro Universitario de Ciencias Exactas e Ingenierías (CUCEI), Universidad de Guadalajara, 44430 Guadalajara, Jalisco Mexico; 2 Facultad de Telemática, Universidad de Colima, 28040 Colima, Colima Mexico; 3 Epidemiology and Biostatistics and Statistics and Probability Departments, Michigan State University, 909 Fee Road, East Lansing, MI 48824 USA; 4 Biometrics and Statistics Unit, International Maize and Wheat Improvement Center (CIMMYT), Apdo. Postal 6-641, 06600 Mexico City, Mexico

**Keywords:** Hyperspectral data, Functional regression analysis, Bayesian functional regression, Functional data, Bayesian Ridge Regression

## Abstract

**Background:**

Modern agriculture uses hyperspectral cameras with hundreds of reflectance data at discrete narrow bands measured in several environments. Recently, Montesinos-López et al. (Plant Methods 13(4):1–23, 2017a. 10.1186/s13007-016-0154-2; Plant Methods 13(62):1–29, 2017b. 10.1186/s13007-017-0212-4) proposed using functional regression analysis (as functional data analyses) to help reduce the dimensionality of the bands and thus decrease the computational cost. The purpose of this paper is to discuss the advantages and disadvantages that functional regression analysis offers when analyzing hyperspectral image data. We provide a brief review of functional regression analysis and examples that illustrate the methodology. We highlight critical elements of model specification: (i) type and number of basis functions, (ii) the degree of the polynomial, and (iii) the methods used to estimate regression coefficients. We also show how functional data analyses can be integrated into Bayesian models. Finally, we include an in-depth discussion of the challenges and opportunities presented by functional regression analysis.

**Results:**

We used seven model-methods, one with the conventional model (M1), three methods using the B-splines model (M2, M4, and M6) and three methods using the Fourier basis model (M3, M5, and M7). The data set we used comprises 976 wheat lines under irrigated environments with 250 wavelengths. Under a Bayesian Ridge Regression (BRR), we compared the prediction accuracy of the model-methods proposed under different numbers of basis functions, and compared the implementation time (in seconds) of the seven proposed model-methods for different numbers of basis. Our results as well as previously analyzed data (Montesinos-López et al. 2017a, 2017b) support that around 23 basis functions are enough. Concerning the degree of the polynomial in the context of B-splines, degree 3 approximates most of the curves very well. Two satisfactory types of basis are the Fourier basis for period curves and the B-splines model for non-periodic curves. Under nine different basis, the seven method-models showed similar prediction accuracy. Regarding implementation time, results show that the lower the number of basis, the lower the implementation time required. Methods M2, M3, M6 and M7 were around 3.4 times faster than methods M1, M4 and M5.

**Conclusions:**

In this study, we promote the use of functional regression modeling for analyzing high-throughput phenotypic data and indicate the advantages and disadvantages of its implementation. In addition, many key elements that are needed to understand and implement this statistical technique appropriately are provided using a real data set. We provide details for implementing Bayesian functional regression using the developed genomic functional regression (GFR) package. In summary, we believe this paper is a good guide for breeders and scientists interested in using functional regression models for implementing prediction models when their data are curves.

**Electronic supplementary material:**

The online version of this article (10.1186/s13007-018-0314-7) contains supplementary material, which is available to authorized users.

## Background

High-throughput phenotyping (HTP) technologies can generate large volumes of data. Many of the phenotypes collected with HTP technologies are high-dimensional. These data can often be represented as functions. Functional data analysis (FDA) is a field of study that deals with the analysis and theory of data whose units of observation are functions (curves) defined in any continuous domain [19]. For instance, one can measure the growth of an organism over time and conceptualize the observed points as (noisy) evaluations of a growth function. Likewise, hyperspectral reflectance (as well as other techniques involving transmittance or absorbance) data can also be thought of as evaluations of a function observed at a sample of points, for example, a number of bands ranging from 392 nm (nm) to 1850 nm [1, 17, 18].

Hyperspectral image data have become increasingly available in agriculture. This information is commonly used to build secondary traits (vegetative index) that are related to primary traits of interest, such as grain yield. For example, in South Australia, hyperspectral data are used to discriminate among grape cultivars [2, 14]. Other applications use hyperspectral data to predict the chemical composition of plants [9], which can be used to detect the nutrient and water status of wheat in irrigated systems [28]. Likewise, infrared spectroscopy is routinely used by the dairy industry in developed countries and the information obtained is used to predict milk components, and health and reproductive outcomes [6].

Recently, we used functional regression analysis (FRA) to develop prediction equations for yield and other traits using hyperspectral crop image data [17, 18]. Our results showed that FRA can provide yield predictions with similar and, in some cases, higher predictive power than that of conventional regression techniques.

FRA and functional analysis use linear combinations of basis functions as the main method to represent functions. The use of basis functions is a computational device well adapted for storing information about functions, since it is very flexible and has the computational power to fit even hundreds of thousands of data points. Moreover, it allows the required calculations to be expressed within the familiar context of matrix algebra [24].

The basic philosophy of FRA and functional data analysis is to think of observed data functions as single entities, rather than merely as a sequence of individual observations. The term functional in reference to observed data refers to the intrinsic structure of the data rather than to their explicit form. In practice, functional data are usually observed and recorded discretely as *m* pairs $(t_{j} , x_{j} )$, and $x_{j}$ is a snapshot of the function at time *t*_*j*_, most of the time blurred by measurement error, but we assume the existence of a smooth function *f* that gave rise to the observed data. Time is very often the continuum over which the functional data are recorded, but other continua, such as wavelength, spatial position, frequency and weight may be involved. A smooth function allows a pair of adjacent data values, $x_{j}$ and $x_{j + 1}$, to be linked together to some extent, since they are unlikely to be too different from each other. If this smoothness property did not apply, there would be nothing much to be gained by treating the data as functional rather than just multivariate [24]. Those interested in deeply understanding the theory and applications of FRA and functional data analysis should read books recently published by Hsing and Eubank [12], Horváth and Kokoszka [13] and Ferraty and Romain [7]. However, the book by Ramsay and Silverman [24], whose first edition was published in 1997, must be cited as a major landmark in the history of functional data analysis. The book by Ferraty and Vieu [8] represents a second-generation view of this subject.

The functional data analysis in general and FRA are being used in many applications (climatology, remote sensing, linguistics, precision agriculture, etc.) where the data are gathered by observing a continuous phenomenon over time or space; see Ramsay and Dalzell [22] for examples. Real-time applications of FRA and functional data analysis can also be found in Rice [21], Müller [20], González-Manteiga and Vieu [10], among others. Henceforth, the improved performance of measurement instruments will make it possible to collect these data on dense grids. They can no longer be considered variables taking values in *R*^*p*^ (as required in conventional statistical methods).

In this article, we provide a brief review of FRA, highlight important aspects of model specification, discuss how FRA can be integrated into Bayesian models [4] and illustrate the application of FRA using real data from high-throughput phenotypic experiments.

## Methods

### Functional regression

FRA is the area of functional data analysis with most applications and methodological developments. FRA can be classified into three types: (a) functional predictor regression (scalar-on-function), where the response variable is scalar and the predictor is a function; (b) functional response regression (function-on-scalar), where the response variable is a function and the predictor a scalar; and (c) function-on-function regression, where both the response and the predictor are functions. In this paper, we will focus only on the first type of FRA. For illustrative purposes, below we describe a model of the first type that contains in the predictor a functional term that represents the information of the curves.

1 $$y_{i} = \int {x_{i} \left( t \right)\beta \left( t \right)dt + e_{i} ,}$$

Here the response variable (*y*_*i*_) is a scalar response of the *i*th observation with $i = 1,2, \ldots ,n$; however, the predictors or covariates are now functions instead of scalar. $x_{i} \left( t \right)$ is the functional predictor and represents the value of a continuous underlying process evaluated at time *t*; unfortunately, in practice the whole curves are not available because they are measured in *m* discretization sample of points $t_{1} < \cdots < t_{m}$ in time or another domain. This means that we only observe discrete noisy trajectories

2 $${\text{x}}_{\text{i}} \left({\text{t}}_{\text{m}} \right) = \text{f}_{\text{i}} \left( {\text{t}}_{\text{m}}\right) + \epsilon_{\text{i}}$$

where $\epsilon_{i}$ is interpreted as random measurement errors (instrument error, human errors,…) at the finite discretization points with a Gaussian distribution with zero mean and variance $\sigma_{ \in }$. Equation (2) is exactly the model proposed by Welham et al. [30] and Verbyla et al. [29] for modeling smoothing splines as mixed models, but this reformulation of Eq. (2) as a mixed model is only possible for cubic smoothing splines.

**Fig. 1:**
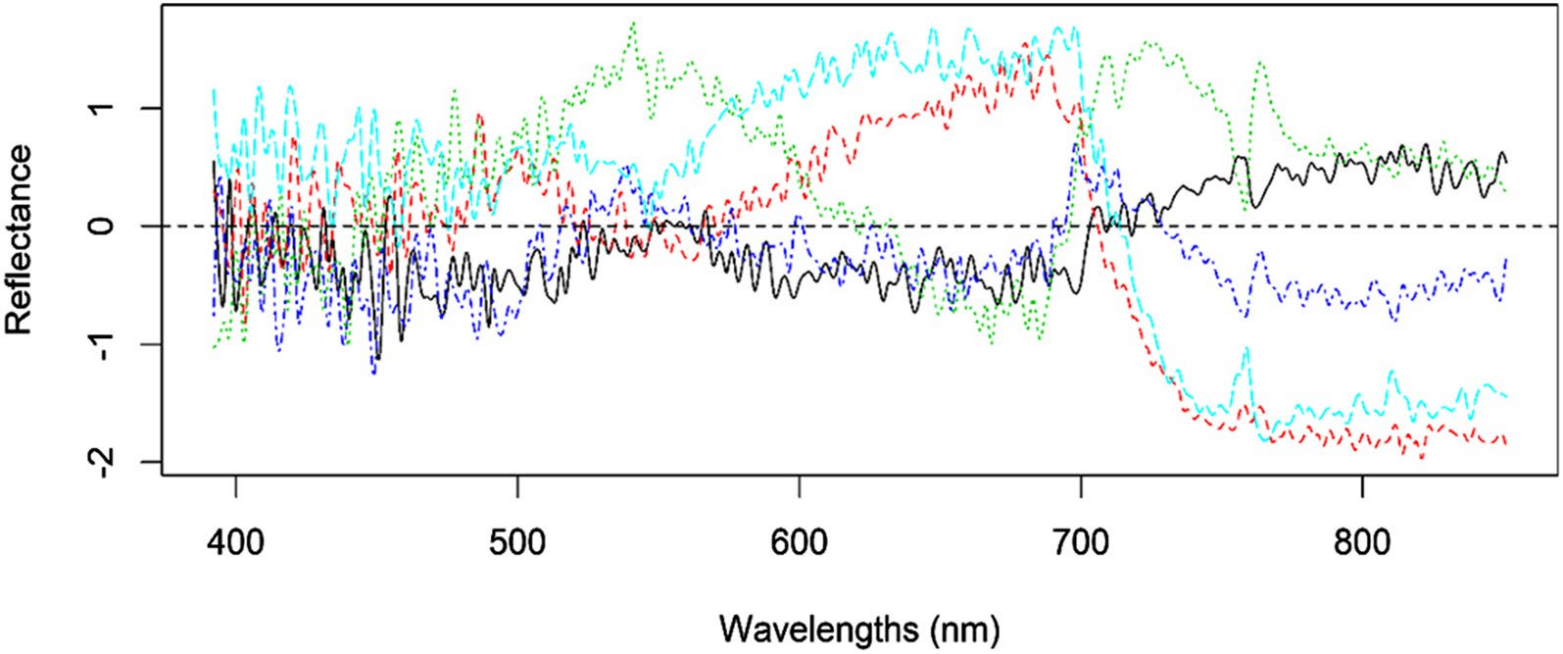
Reflectance (centered to a zero mean) measured over 250 wavelengths in the 392 to 850 nm range of the light spectrum. Each curve corresponds to data of a maize genotype planted in an irrigated environment and measured at Cd. Obregón, Mexico

In functional regression (of scalar-on-function type), this model (Eq. 2) is used to smooth each row (curve) of the predictor information given in Eq. (1), since in this context each row represents a curve and a curve (called a datum) is not a single observation, but rather a set of measurements along a continuous domain, which, taken together, should be regarded as a single entity [15]. Hyperspectral images are an example of functional data (predictor information) obtained on the reflectance of electromagnetic power on large numbers of wavelengths, that is: $\varvec{x}_{i} = \left( {x_{i1} ,x_{i2} , \ldots ,x_{im} } \right)'$. Here, *x*_*it*_ represents the reflectance observed at the tth wavelength on the *i*th sample (e.g., genotype). The first goal is to infer $f_{i} \left( {t_{m} } \right)$. This can be achieved using smoothing techniques [26, 27]. Figure 1 illustrates this approach. Reflectance is represented on the vertical axis and wave numbers (in the 392 to 850 nm range) are represented on the x-axis. The different curves correspond to different genotypes and each curve represents a datum.

In Fig. 1 the unknown function, ${\text f}_{{\text i}} \left( {{\text t}_{{\text m}} } \right)$, is represented as a linear combination of a set of L basis functions, $\phi_{il} \left( {t_{m} } \right)$, that are non-linear functions of the input variable (*t*_*m*_), that is, $f_{i} \left( {t_{m} } \right) = \mathop \sum \nolimits_{l = 1}^{L} c_{il} \phi_{il} \left( {t_{m} } \right)$; here $c_{il}$ are regression coefficients that can be estimated by regressing *x*_*im*_ on the basis functions, i.e., by fitting $x_{im} = \sum\nolimits_{l = 1}^{L} {c_{il} \phi_{il} \left( {t_{m} } \right) + \varepsilon_{im} .}$ Some of the most popular basis functions include the Fourier and splines basis.

#### Fourier basis

A Fourier series is an expansion of a periodic function *f*(*t*) in terms of an infinite sum of sines and cosines that are orthogonal. According to Reig et al. [25], the study and computation of a Fourier series (called harmonic analysis) is often useful as a way to break up an arbitrary periodic functions into a set of simple terms that can be solved individually and then recombined to obtain the solution to the original problem, or an approximation of it, to whatever accuracy is desired or practical.

#### Splines

Splines are piece-wise polynomials fitted within intervals defined by a set of knots; they tend to be used to describe functional information without any strong cyclic variation. The elements that define a spline are: the family or type (e.g., B-splines), the degree of polynomials used to build the spline (linear, quadratic, cubic, etc.) and the set of knots that define the bins within which the polynomials are fitted. The basis functions of a spline are defined in such a way that the function is continuous and has continuous derivatives everywhere (including at the knots) of order q-1; here q is the order of the polynomial. The number of basis functions (*L*) in a B-spline is *L *=* q *+ *1 *+* K*, where *K* denotes the number of interior knots [3].

**Fig. 2:**
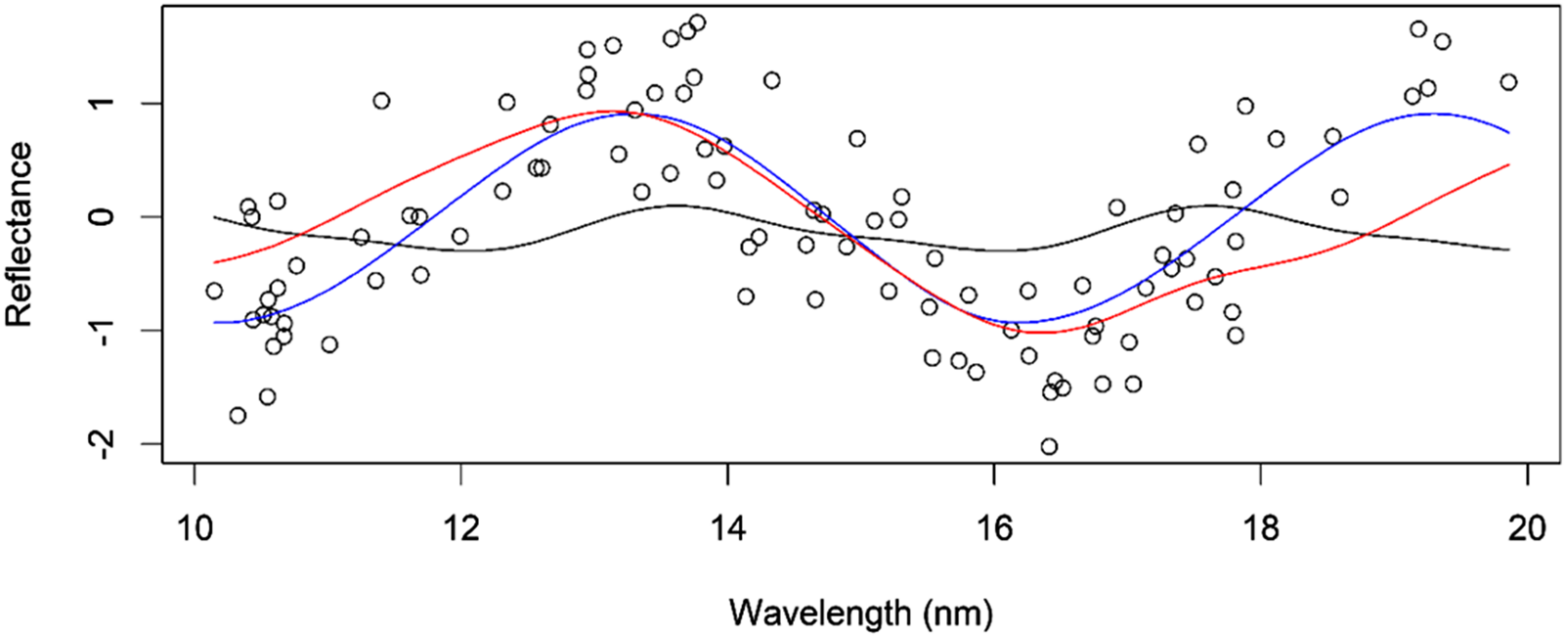
Scatterplot of the hypothetical phenomenon. The dots represent the 100 data points measured. This smoothing plot was done using *L *= 11 basis for three values of the period *T *= 4 (black color), 6 (blue color) and 8 (red color)

**Fig. 3:**
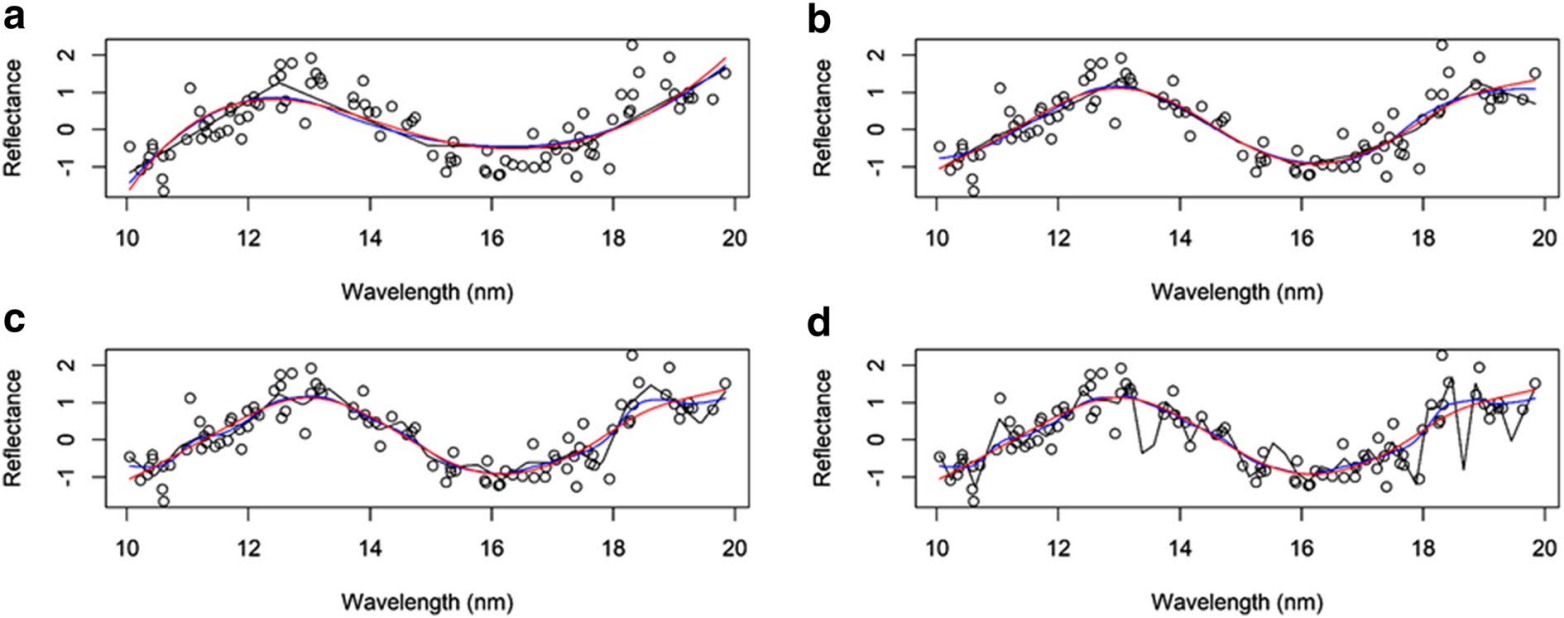
Scatterplot of the hypothetical phenomenon. The dots represent the 100 data points measured: **a** this smoothing plot was done using *L *= 5 basis for three values of the B-spline with degree 1 (linear; black color), 2 (quadratic; red color) and 3 (cubic; blue color); **b** this smoothing plot was done using *L *= 11 basis for three values of the B-spline with degree 1, 2 and 3; **c** this smoothing plot was done using *L *= 25 basis for three values of the B-spline degree 1, 2 (quadratic) and 3 (cubic); **d** this smoothing plot was done using *L *= 51 basis for three values of the B-spline degree 1, 2 and 3

The process of using basis functions is shown in Figs. 2 and 3. In both cases, the unknown function was sine $f\left( {t_{k} } \right) = \sin \left( {1 + t_{k} } \right)$, the set of points $\left\{ {t_{k} } \right\}$ were drawn from a uniform distribution in the interval between 10 and 20, and errors were drawn from a normal distribution with mean 0 and a standard deviation of 0.5. In Fig. 2, we approximated the function using the Fourier basis. Here, we considered using L = 11 basis functions and three different values for the period (T = 4, 6 and 8). The approximation (representation) of the curve is very poor for periods *T *= 4 and 8. However, for *T *= 6, the representation of the curve is good. It is important to point out that to make Fig. 2, we changed the number of basis (5, 25 and 51) but did not find any difference when using L = 11 in Fig. 2. In Fig. 3, we approximated the function using a spline with L = 5, 11, 25 and 51 basis and we considered linear, quadratic and cubic splines. Using *L* = 5 basis is not enough to reasonably represent the curve (Fig. 3a). However, when *L *= 11, 25 and 51 basis, the smoothing curves do a reasonable job for quadratic and cubic splines. However, the performance of the linear spline was not good and using *L *= 51 yields overfitting.

The examples presented in Figs. 2 and 3 highlight the importance of carefully choosing the type of basis function and the value of the period for the Fourier basis. In general the main elements of model specification in FRA and functional data analysis include the choice of the family of basis functions (e.g., splines or Fourier) and parameters that may index each family (e.g., knots in a spline), the number of basis functions (or model degrees of freedom), and the method used to estimate regression coefficients (e.g., least squares).

In similar fashion, $\beta \left( t \right)$ in Eq. (1) is the beta functional regression coefficient and *e*_*i*_ is an error term assumed normal with mean zero and variance $\sigma^{2}$. Several methods can be used to reconstruct the functional form of the sample paths from the observed discrete data, depending on: (a) the manner in which these data were obtained in discrete time, and (b) the way we expect the curve to behave.

#### Conventional functional regression with scalar response and functional predictor

It is usually assumed that the sample trajectories $x_{i} \left( t \right)$ that appear in Eq. (2) belong to a finite-dimensional space generated by a truncated basis $\phi_{1} \left( t \right), \ldots ,\phi_{L} \left( t \right)$ and is expressed as

3 $$x_{i} \left( t \right) = c_{i1} \phi_{1} \left( t \right) + c_{i2} \phi_{2} \left( t \right) + \ldots + c_{iL} \phi_{L} \left( t \right),$$

where *L* represents the number of basis functions, and $\phi_{l} (t$) is the *l*th basis function evaluated in *t*. The basis functions $\phi_{l} (t$) are a system of functions specially chosen to be used as building blocks that represent a smooth curve. There are many different types of basis function systems, as mentioned above. $c_{il}$ is the basis coefficient corresponding to the *i*th individual of the $\phi_{l} (t$) function and determines the relative weights of each basis function when constructing the curve for datum *i*. Assuming that each curve was observed in $\varvec{t} = [t_{1} , \ldots , t_{m} ]^{T}$, then in vector form

$$x_{i} \left( \varvec{t} \right) = \left[ {\begin{array}{*{20}c} {\mathop \sum \nolimits_{l = 1}^{L} c_{il} \phi_{l} \left( {t_{1} } \right)} \\ \vdots \\ {\mathop \sum \nolimits_{l = 1}^{L} c_{il} \phi_{l} \left( {t_{m} } \right)} \\ \end{array} } \right] = \left[ {\begin{array}{*{20}c} {\phi_{1} \left( {t_{1} } \right)} & \cdots & {\phi_{L} \left( {t_{1} } \right)} \\ \vdots & \ddots & \vdots \\ {\phi_{1} \left( {t_{m} } \right)} & \cdots & {\phi_{L} \left( {t_{m} } \right)} \\ \end{array} } \right]\varvec{c}_{i} = {\varvec{\Phi}}\varvec{c}_{i}$$

where $\varvec{c}_{i}^{T} = \left[ {c_{i1} , \ldots ,c_{iL} } \right]$ of order 1 × *L*. Therefore, the values of $\varvec{c}_{i}$ that best represent $x_{i} \left( \varvec{t} \right)$ in terms of minimizing $\left[ {x_{i} \left( \varvec{t} \right) - {\varvec{\Phi}}\varvec{c}_{i} } \right]^{T} \left[ {x_{i} \left( \varvec{t} \right) - {\varvec{\Phi}}\varvec{c}_{i} } \right]$ are given by

4 $$\hat{\varvec{c}}_{i} = \left[ {{\varvec{\Phi}}^{T} {\varvec{\Phi}}} \right]^{ - 1} {\varvec{\Phi}}^{T} x_{i} \left( \varvec{t} \right)$$

In other words, Eq. (4) used the least-square method to produce the smoothed estimates by multiplying the raw observations by the “smoother” or “hat” matrix $H = \left[ {{\varvec{\Phi}}^{T} {\varvec{\Phi}}} \right]^{ - 1} {\varvec{\Phi}}^{T} ,$ where ${\varvec{\Phi}}$ is the matrix of basis functions of order *m *× *L*. Assuming that the beta functional coefficients given in Eq. (1) can be expressed as a linear combination of a truncated basis, $\psi_{1} \left( t \right)$, …, $\psi_{S} \left( t \right);$ since $\beta \left( t \right) = \mathop \sum \nolimits_{s = 1}^{S} d_{s} \psi_{s} \left( t \right)$, the model given in Eq. (1) can be rewritten as

5 $$y_{i} = \mathop \sum \limits_{s = 1}^{S} d_{s} \smallint x_{i} \left( t \right)\psi_{s} \left( t \right)dt + e_{i} = \mathop \sum \limits_{s = 1}^{S} d_{s} w_{is} + e_{ij} = \varvec{w}_{i}^{T} \varvec{d} + e_{i} ,$$

where $w_{is} = \smallint x_{i} \left( t \right)\psi_{s} \left( t \right)dt$, $\varvec{w}_{i}^{T} = \left[ {w_{i1} , \ldots ,w_{iS} } \right],$ and $\varvec{d}^{T} = \left[ {d_{1} , \ldots ,d_{S} } \right]$ is an unknown vector of coefficients related to the effect of the functional covariate. Substituting the obtained representation of $x_{i} \left( t \right)$ in $w_{is} = \smallint x_{i} \left( t \right)\psi_{s} \left( t \right)dt$, the elements of $\varvec{w}_{i}^{T}$ can be explicitly approximated as

6 $$w_{is} = \int {x_{i} \left( t \right)\psi_{s} \left( t \right)dt} = \mathop \sum \limits_{l = 1}^{L} \hat{c}_{il} \smallint \phi_{l} \left( t \right)\psi_{s} \left( t \right)dt,$$

where the coefficients $\hat{c}_{il}$ are given in Eq. (4). Then, by making $J_{ls} = \smallint \phi_{l} \left( t \right)\psi_{s} \left( t \right)dt$, the $\varvec{w}_{i}^{T}$ can be computed in vector form as

7 $$\varvec{w}_{i}^{T} = \left[ {\begin{array}{*{20}c} {\mathop \sum \nolimits_{l = 1}^{L} \hat{c}_{il} J_{l1} } \\ \vdots \\ {\mathop \sum \nolimits_{l = 1}^{L} \hat{c}_{il} J_{lS} } \\ \end{array} } \right]^{T} = \left[ {\begin{array}{*{20}c} {\hat{c}_{i1} J_{11} } & \cdots & {\hat{c}_{i1} J_{1S} } \\ \vdots & \ddots & \vdots \\ {\hat{c}_{iL} J_{L1} } & \cdots & {\hat{c}_{iL} J_{LS} } \\ \end{array} } \right] = \hat{\varvec{c}}_{i}^{T} \left[ {\varvec{J}_{1} \ldots \varvec{J}_{S} } \right] = \hat{\varvec{c}}_{i}^{T} \varvec{ J} = x_{i}^{T} \left( \varvec{t} \right){\varvec{\Phi}}\left[ {{\varvec{\Phi}}^{T} {\varvec{\Phi}}} \right]^{ - 1} \varvec{J}$$

where $\varvec{J}_{s} = \left[ {J_{1s} , \ldots ,J_{Ls} } \right]^{T}$, $\varvec{J} = \left\{ {J_{ls} } \right\}$ is of order $L \times S$ and $\hat{\varvec{c}}_{i}^{T} = \left[ {\hat{c}_{i1} , \ldots ,\hat{c}_{iL} } \right]$. When the same basis functions are used for *x*(*t*) and $\beta \left( t \right)$, and *L *= *S*, then $\varvec{J} \approx {\varvec{\Phi}}^{T} {\varvec{\Phi}}$ (see Additional file 1: Part B-SB3). Therefore, since we obtained $\varvec{w}_{i}^{T}$ by stacking the *n* rows corresponding to each $\varvec{w}_{i}^{T} ,$ and since $i = 1,2, \ldots ,n$, we formed the matrix $\varvec{W} = [\varvec{w}_{1}^{T} ,..,\varvec{w}_{n}^{T} ]^{T} = \varvec{X}{\varvec{\Phi}}\left[ {{\varvec{\Phi}}^{T} {\varvec{\Phi}}} \right]^{ - 1} \varvec{J}$, with $\varvec{X} = [\varvec{x}_{1}^{T} \left( \varvec{t} \right),..,\varvec{x}_{n}^{T} \left( \varvec{t} \right)]^{T}$, which allows implementing the functional regression model given in Eq. (1) using conventional Bayesian or classic modeling (see [23, 24] for more details and considerations). The functional regression given in Eq. (1) can be written in vector form as the following linear model:

8 $$\varvec{Y} = \varvec{Wd} + \varvec{E}$$

where $\varvec{Y} = \left( {y_{1} , \ldots ,y_{n} } \right)^{T}$, the vector of response variables (grain yield), ***d*** are the beta coefficients of order *S *× 1 associated with the representation of $\beta \left( t \right)$ in terms of the truncated basis $\psi_{1} \left( t \right)$, …, $\psi_{S} \left( t \right)$, and $\varvec{E} = \left( {e_{1} , \ldots ,e_{n} } \right)^{T}$ is a vector of errors of dimension *n *× 1. In the implementations given in the next section, we will use *S *= *L*.

#### Alternative models of the functional regression model with scalar response and functional predictor

As an approximation of the functional regression given in Eq. (1), we can regress the vector of response variable ***Y*** against the approximate design matrix of curves, $\varvec{X}^{*} ,$ which results in the following traditional linear model:

9 $$\varvec{Y} = \varvec{X}^{*}\varvec{\beta}_{*} + \varvec{E}$$

It is important to point out that ***X*** and $\varvec{X}^{*}$ are both of order *n *× *m*, but of column rank *m* and L, respectively (where L is the number of basis) (see Appendix A for details on how to derive this model). $\varvec{\beta}_{*}$ are beta coefficients of order *m *× 1 and ***E*** is a vector of errors as previously defined. This approximation (Eq. 9) of the functional regression model given in Eq. (1) does not provide any gain in terms of implementation time compared with directly regressing the vector of response variable ***Y*** against the original design matrix ***X***, since the beta coefficient required is exactly *m*, as under the original design matrix. However, for prediction purposes, we can reparametrize the model given in Eq. (9) as:

10 $$\varvec{Y} = \varvec{X}^{**}\varvec{\beta}_{**} + E$$

where $$\varvec{X}^{**} = \varvec{X}{\varvec{\Phi}}$$ and $$\varvec{\beta}_{**} = \left[ {{\varvec{\Phi}}^{T} {\varvec{\Phi}}} \right]^{ - 1} {\varvec{\Phi}}^{T}\varvec{\beta}_{*}$$. Now X^**^ is of order *n *× *L* and $$\varvec{\beta}_{**}$$ of order *L *× 1 (for details on how Eq. 10 was derived, see Appendix A). The advantage of working with Eq. (10) as compared to working with Eq. (9) is two-fold: (a) it is numerically more stable when estimating the parameters; and (b) it reduces the dimensionality from *m* to *L*, which implies that fewer beta coefficients need to be estimated assuming that *L *< *m*; now the design matrix X^**^ is full column rank, and we do not need ***X*** when estimating the equations, which is advantageous because ***X*** has many columns and is often not full column rank. It is important to point out that the proposed alternative given in Eq. (10) is similar to the alternative proposed by Marx and Eilers [16], but with the main difference that they arrived at this alternative by smoothing only the beta coefficients, while we arrived at it by smoothing the ***X*** matrix. The parameterization given in Eq. (10) for fitting a functional regression model should be attractive when *L *< *m* because it will reduce the dimensionality of the regression problem considerably, improve implementation time and produce more stable parameter estimates.

### Elements for modeling functional data

Good performance of the functional regression model strongly depends on choosing the right type of basis functions, the required number of basis, the degree of the polynomial (for B-splines), knot locations (for B-splines), the period (in Fourier basis), and others. Next we provide some practical rules that can help researchers and practitioners select all the necessary tuning parameters more efficiently.

#### Basis function expansion

In FRA, we have functional objects as predictor variables rather than sample points. Therefore, the discrete data need to be converted into a smooth functional object. However, before we can convert raw discrete samples into a functional data object, we must specify a system of basis functions that consists of simple smooth functions that are linearly combined to approximate actual functions with an arbitrary level of accuracy. Here we replace observations ($x_{i} , i = 1, \ldots ,m$, data points) with $x\left( {t_{i} } \right),$ where *x*(*t*) is a smooth function formed by a linear combination of basis functions, as shown in Eq. (3). However, it is important to be aware that there are many options for basis functions. Two of the most popular basis functions are the Fourier and B-spline basis functions. Other popular basis functions are polynomial basis functions, Gaussian basis functions, radial basis functions, wavelet basis functions, and orthogonal basis functions. In general, the choice of basis functions depends on the nature of the signal; for this reason, one may prefer a Fourier series to summarize cyclic, seasonal trends in data. On the other hand, B-splines are not restricted to being periodic and often provide flexibility for modeling deviations from seasonal trends. B-splines are also computationally efficient as they have compact support; any B-spline function is only non-zero over a range of a small number of distinct knots [11], while wavelet basis are more suitable for sharp local features like heart rate. For this reason, it is of paramount importance to experiment with different alternative basis functions, numbers of basis, periods, degrees of the basis, etc.

#### Degrees of freedom and interval period

The degrees of freedom is a tuning parameter to be selected in B-spline. However, the most common choices are 1, 2 or 3 for computational convenience, but degree three is most often used because in general it does a good job in terms of the quality of the fit and implementation time [5].

#### Knot location and knot number selection

For B-splines, the knot location and the selection of the number of knots are crucial factors to guarantee good performance by the regression spline smoother. Two widely used methods for locating the knots are: (a) the uniform knot-spacing method, and (b) Quantiles as a knots method. Two methods for selecting the number of knots are: (a) the generalized cross-validation (GCV) method, and (b) the coefficient of determination (*R*^2^). These four methods are explained in detail in Additional file 1: Part B-SB1.

#### Data set

Seven methods (see Table 1) were implemented using a data set that consisted of 976 wheat lines that were evaluated for grain yield (GY) in an irrigated environment and measured on March 17, 2015; this data set is part of the data set used by Montesinos-López et al. [17, 18]. In each plot for each wheat line, 250 wavelengths $$\lambda_{1} , \ldots \lambda_{250}$$ were measured from 392 to 851 nm on the light spectrum. The *k*th discretized spectrometric curve of a given genotype is given by $$x_{1} \left( {\lambda_{1} } \right), \ldots ,x_{250} \left( {\lambda_{250} } \right)$$. We used the notation $$x\left( {780} \right)$$ without subscripts to denote the response of the band measured at 780 wavelengths, $$x\left( {670} \right)$$ to denote the response of the band measured at 670 wavelengths, and so on. The trait of interest GY (which we want to predict) and the 250 bands are best linear unbiased estimations (BLUEs) of the 976 genotypes obtained in a first pass analysis that takes into account the design effect; details of how they were obtained can be found in Montesinos-López et al. [17, 18]. More details about this data set can be found in Montesinos-López et al. [17, 18]. With the information on these 250 bands, we formed the design matrix **X** presented in Table 1; the other design matrices ($$\varvec{W},\varvec{X}^{*} , \varvec{X}^{**} )$$ shown in Table 1 were created based on this information. Since our goal is to obtain predictions of missing phenotypes on some genotypes taking into account the hyperspectral image data, to assess the prediction accuracy of the seven methods, we implemented a type of cross-validation that mimics a situation where the researcher wants to predict 33.33% of the lines in a specific environment. For this reason, to study the prediction accuracy of the sample data, a threefold cross-validation was implemented with twofold for training and onefold for testing. Then, for each fold, we fitted the models using the training data set, and with the testing data set, we evaluated the prediction performance using Pearson’s correlation. The averages of the threefolds are reported together with their standard error as a measure of prediction accuracy.

**Table 1:** Methods proposed, predictors, basis type and type of model

Method	Predictor of the model	Basis type	Type of model
M1	**X**	None	Conventional regression
M2	***W***	B-splines	Functional regression (Eq. 8)
M3	***W***	Fourier	Functional regression (Eq. 8)
M4	***X*** ^*^	B-splines	Alternative 1 for Functional regression (Eq. 9)
M5	***X*** ^*^	Fourier	Alternative 1 for Functional regression (Eq. 9)
M6	***X*** ^**^	B-splines	Alternative 2 for Functional regression (Eq. 10)
M7	***X*** ^**^	Fourier	Alternative 2 for Functional regression (Eq. 10)

Grain yield (***Y***) is the vector response of the variable (trait of interest)

### The proposed framework

In this manuscript, we provide a framework for implementing Bayesian functional regression models that are better suited for use in a prediction context. The framework given is flexible enough to construct functions from noisy discrete data sets, and reasonably easy and fast in terms of implementation since the developed R package that we called Genomic Functional Regression (GFR) (Additional file) can be used. The GFR package is able to implement conventional regression models and functional regression models (of the scalar-on-function type) for normal, binary and ordinal data with various alternative shrinkage methods (Bayesian Ridge regression (BRR), Bayes A (BayesA), Bayes B (BayesB), Bayes C and Bayesian Lasso (BL)) under a Bayesian framework in the context of genomic selection when tens or hundreds of thousands of data are available. It is important to point out that the developed GFR package was built based on the BGLR package [4]. For illustrative purposes, we propose implementing seven methods (Table 1). The assumptions of the prior distributions used for implementing the seven proposed models given in Table 1 are provided in Additional file 1: Part B-SB2.

#### R code for implementing the proposed functional regression models

The R code for implementing the seven proposed methods with the GFR package is given in Appendix B; for specific details on how to install the GFR package, see Additional file 1: Part A. The reader can modify this code slightly and use it with his/her own data. Also, if the researcher wants to include more elements in the linear predictor (such as main effects of lines, environment, genotype by environment interaction, bands and the bands by environments interaction term), we suggest reading the article by Montesinos-López et al. [17, 18], which describes many different ways of specifying the linear predictor including genomic and pedigree information, as well as consulting Additional file 1: Part A, where we provide other examples for using the developed package.

## Results

### Application example

To illustrate the modeling process and compare the prediction accuracy of the seven proposed methods using functional regression in the context of high-throughput phenotyping data, we used the data set described above (irrigated data). First, under a BRR model, we compared the prediction accuracy (with Pearson’s correlation between predicted and observed values) of the methods proposed under different numbers of basis functions. These seven proposed methods resulted from the implementation of the three models provided for functional regression analysis under different priors for the beta coefficients. Also, under the BRR model, we compared the implementation time (in minutes) of the seven proposed methods for different numbers of basis. Next, we compared methods M3, M5 and M7 under different period values (*T*) to examine the impact on prediction accuracy of selecting different values of *T*. Finally, we compared the prediction accuracy of the seven proposed methods under three types of regularization methods using three numbers of basis (5, 29 and 51).

#### Prediction accuracy under different numbers of basis functions

**Fig. 4:**
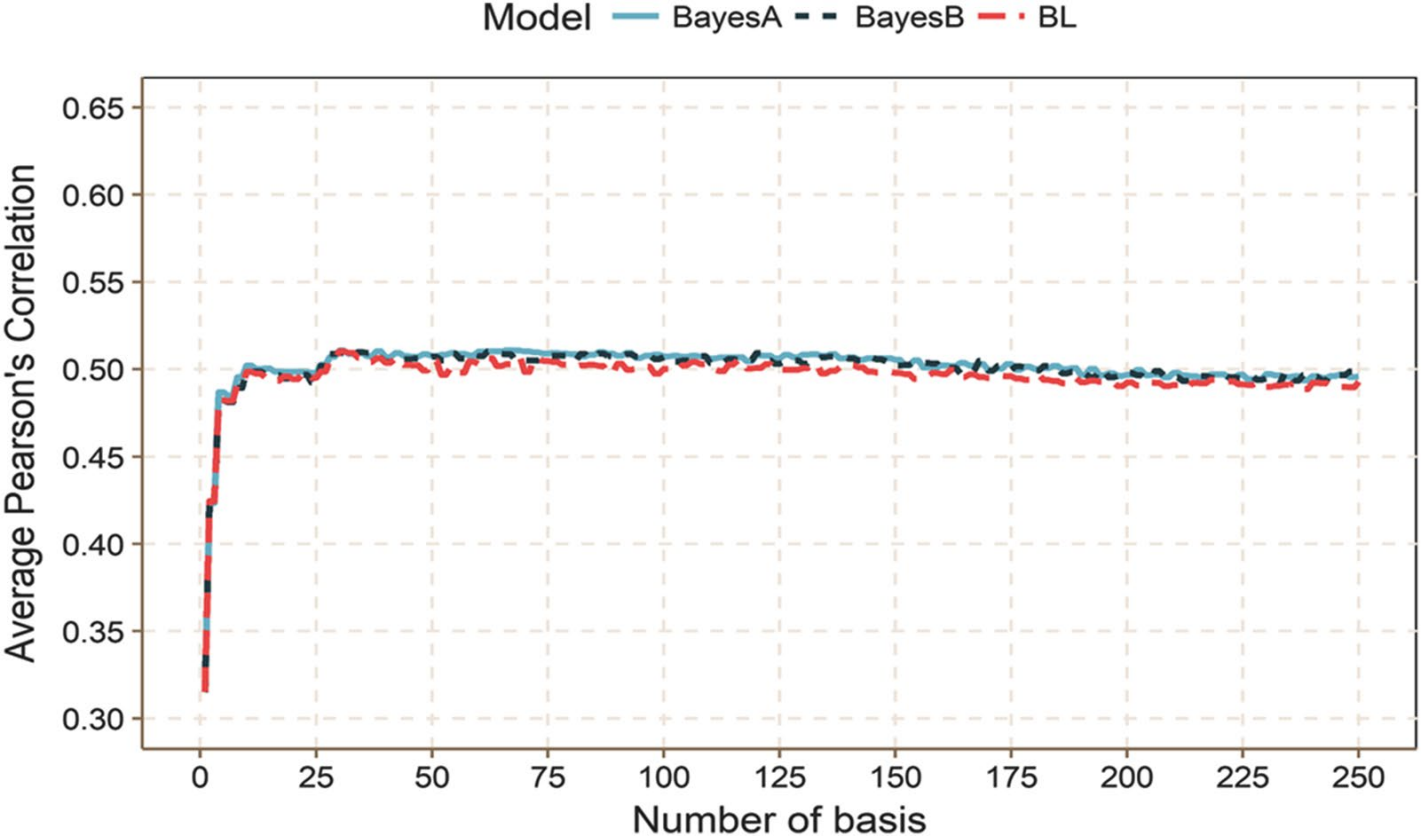
Optimal number of basis (*L*) for method 7 with three regularization methods

First we compared the prediction accuracy of the seven proposed methods for each number of basis and did not find significant differences between methods for each number of basis with an analysis of variance (ANOVA) and the Tukey test. This means that, statistically, the seven methods have the same performance in terms of prediction accuracy for each number of basis (Table 2). We also compared the prediction accuracies for each method between the number of basis and according to the ANOVA and Tukey test at a 5% level of significance; we also did not find statistical differences in terms of prediction accuracy between the number of basis for each method under study (Table 2). On the other hand, Fig. 4 shows that when the number of basis (L) is less than 13, the predictions are lower. For this data set, the best predictions were observed between 13 and 200 number of basis and Fig. 4 shows that after 200 basis, the prediction accuracy starts to decrease. No problems of overfitting were observed in the range of number of basis examined in this study, even with 200 to 250 basis.

**Table 2:** Prediction accuracy of grain yield with Pearson’s correlation for the 7 proposed methods with BRR prior distribution for different numbers of basis functions

Method	Parameter	Number of basis																											Average	
		5			11			17			23			29			35			41			45			51				
					Pearson’s correlation																									
M1	Mean	0.494	a	A	0.494	a	A	0.494	a	A	0.494	a	A	0.494	a	A	0.494	a	A	0.494	a	A	0.494	a	A	0.494	a	A	0.494	a
	SE	0.023			0.023			0.023			0.023			0.023			0.023			0.023			0.023			0.023			0.023	
M2	Mean	0.466	a	A	0.482	a	A	0.494	a	A	0.495	a	A	0.496	a	A	0.498	a	A	0.497	a	A	0.498	a	A	0.497	a	A	0.491	a
	SE	0.020			0.023			0.021			0.024			0.022			0.022			0.022			0.023			0.237			0.022	
M3	Mean	0.482	a	A	0.496	a	A	0.495	a	A	0.494	a	A	0.498	a	A	0.498	a	A	0.498	a	A	0.499	a	A	0.498	a	A	0.495	a
	SE	0.020			0.023			0.023			0.024			0.025			0.025			0.025			0.024			0.025			0.024	
M4	Mean	0.480	a	A	0.495	a	A	0.496	a	A	0.497	a	A	0.496	a	A	0.495	a	A	0.497	a	A	0.496	a	A	0.498	a	A	0.494	a
	SE	0.024			0.025			0.024			0.025			0.025			0.025			0.026			0.026			0.024			0.025	
M5	Mean	0.483	a	A	0.494	a	A	0.495	a	A	0.494	a	A	0.495	a	A	0.500	a	A	0.497	a	A	0.499	a	A	0.498	a	A	0.495	a
	SE	0.021			0.023			0.025			0.024			0.026			0.023			0.025			0.024			0.024			0.024	
M6	Mean	0.466	a	A	0.482	a	A	0.493	a	A	0.495	a	A	0.497	a	A	0.498	a	A	0.497	a	A	0.498	a	A	0.497	a	A	0.491	a
	SE	0.020			0.023			0.022			0.024			0.022			0.022			0.022			0.023			0.024			0.022	
M7	Mean	0.482	a	A	0.496	a	A	0.495	a	A	0.493	a	A	0.498	a	A	0.498	a	A	0.498	a	A	0.499	a	A	0.498	a	A	0.495	a
	SE	0.020			0.023			0.023			0.024			0.025			0.025			0.025			0.024			0.025			0.024	
		Implementation time																												
M1	Mean_T	30.89	a	A	30.89	a	A	30.89	a	A	30.89	a	A	30.89	a	A	30.89	a	A	30.89	a	A	30.89	a	A	30.89	a	A	30.89	a
	SE_T	3.27			3.27			3.27			3.27			3.27			3.27			3.27			3.27			3.27			3.27	
M2	Mean_T	8.4	c	AB	8.9	c	AB	9.32	c	AB	9.51	c	AB	9.34	b	AB	10.1	b	A	10.33	b	A	9.65	b	AB	6.79	c	B	9.150	c
	SE_T	0.22			0.25			0.3			0.13			0.16			0.22			0.41			0.96			0.06			0.67	
M3	Mean_T	7.89	c	AB	8.32	c	AB	8.61	c	AB	9.31	c	AB	9.79	b	AB	10.37	b	A	9.97	b	AB	8.91	b	AB	6.71	c	B	8.879	c
	SE_T	0.19			0.04			0.14			0.33			0.04			0.07			0.52			0.82			0.01			0.7	
M4	Mean_T	27.17	b	A	27.01	b	A	26.83	b	A	26.78	b	A	27.15	a	A	27.16	a	A	27.12	a	A	27.32	a	A	16.68	b	B	25.910	b
	SE_T	0.22			0.11			0.28			0.36			0.63			0.52			0.44			0.5			0.16			1.95	
M5	Mean_T	27.53	b	A	27.4	b	A	27.15	b	A	27.21	b	A	27.12	a	A	27.16	a	A	27.25	a	A	27.27	a	A	16.84	b	B	26.110	b
	SE_T	1.12			1.12			0.83			0.91			0.63			0.67			0.62			0.73			0.3			2.04	
M6	Mean_T	8.03	c	AB	8.5	c	AB	8.68	c	AB	9.12	c	AB	10.19	b	AB	10.59	b	AB	10.91	b	A	10.97	b	A	7.17	c	B	9.350	c
	SE_T	0.04			0.08			0.09			0.07			0.19			0.23			0.05			0.46			0.59			0.79	
M7	Mean_T	7.92	c	AB	8.34	c	AB	8.61	c	AB	9.2	c	AB	9.55	b	AB	9.76	b	AB	10.09	b	A	10.05	b	AB	7.15	c	B	8.970	c
	SE_T	0.08			0.1			0.1			0.07			0.24			0.22			0.31			0.47			0.49			0.61	

Mean is the average Pearson’s correlation and SE is the standard error. Mean_T and SE_T are the average and standard error (in seconds) for implementing each scenario. Average is the average across the number of basis. Different lowercase letters by the columns indicate statistical differences between methods with the Tukey test at 5% level of significance. Different uppercase letters by row indicate statistical differences between numbers of basis with the Tukey test at 5% level of significance

#### Implementation time of the proposed models

Table 2 also shows the performance of the proposed methods in terms of their implementation time (in seconds) under a BRR approach. We found significant differences between the 7 proposed methods in terms of their implementation times according to the ANOVA and Tukey test at the 5% level of significance. Under $L = 5, 11, 17, 23\,{\text{and}}\, 51,$ we found that the best methods were M2, M3, M6 and M7, while the worst was method M1. Under $L = 29, 35, 41 {\text{and }}\, 45,$ the best methods were also M2, M3, M6 and M7, while the worst were the remaining methods: M1, M4 and M5. Finally, for the average across basis we found that the best methods were M2, M3, M6 and M7, while the worst was method M1. It is interesting to point out that among the best methods in terms of implementation time were the two alternative methods (M6 and M7) that used the X^**^ design matrix and had *L* columns.

In general, methods M2, M3, M6 and M7 (all of them used a design matrix with reduced dimensionality) were the best in implementation time. The reduction in time with methods M1, M4 and M5 (that did not use a design matrix with reduced dimensionality) was around 3.4 times, that is, methods M2, M3, M6 and M7 were around 3.4 times faster than methods M1, M4 and M5. Table 2 also shows that there were significant differences in implementation time between the number of basis for all methods, except method M1 because it does not use basis. For example, in methods M2 and M6, the best implementation times were observed with *L *= 51 number of basis, while the worst times were observed with $L = 35\, {\text{and}}\, 41$ basis in M2 and $L = 41\, {\text{and}}\, 45$ in M6. In methods M3 and M7, the best implementation times were also observed with *L *= 51 number of basis, but the worst time was observed with *L *= 35 basis in M3 and *L *= 41 basis in M7. In methods M4 and M5, the best implementation times were observed with *L *= 51 number of basis, and the worst times were observed with the rest of the number of basis. It is important to point out that we were expecting that the lower the number of basis, the lower the implementation time required for most of the proposed methods; however, there was not a large difference in time reduction when using *L *= 5 and *L *= 51. Of course, it is to be expected that if *L* grows close to *m* (original dimension of the data), the implementation time will increase considerably.

#### Prediction accuracy under different numbers of periods for the Fourier basis

Table 3 compares the prediction accuracy of methods M3, M5 and M7 (methods with the Fourier basis) using *L *= 23 (number of basis) and 9 different period values (*T*). We did not find significant differences in terms of prediction accuracy (with Pearson’s correlation) between the 9 periods for each of the three methods (M3, M5 and M7) according to the ANOVA at a 5% level of significance. The minimum prediction observed in the results of Table 3 was 0.4607, while the largest was 0.4936. Since significant differences were not observed in terms of prediction accuracies between the 9 period values under study for each method, we can say that for this dataset, using functional regression with the Fourier basis is quite robust for choosing the value of the period, since the differences between the different values of the test period were not significant. However, it is important to point out that choosing the value of the period depends strongly on the type of data at hand (recall the example given in Fig. 2). For this reason, before implementing a functional regression model, it is very important to experiment with different period values, numbers of basis, types of basis, degrees, etc., to increase the probability of success in the modeling process and subsequent analysis.

**Table 3:** Prediction accuracy of grain yield with Pearson’s correlation for the 7 proposed methods with BRR prior distribution for different numbers of periods for the Fourier basis

Period	M3			M5			M7		
	Mean		SE	Mean		SE	Mean		SE
51	0.4609	a	0.0224	0.4607	a	0.0219	0.4616	a	0.0218
57.38	0.4658	a	0.0211	0.4655	a	0.0213	0.4658	a	0.0214
65.57	0.4639	a	0.0201	0.4623	a	0.0206	0.4636	a	0.02
76.5	0.4706	a	0.0219	0.4666	a	0.0214	0.4705	a	0.0217
91.8	0.4757	a	0.019	0.4755	a	0.0192	0.4755	a	0.0191
114.75	0.4636	a	0.0204	0.4631	a	0.0202	0.4638	a	0.0201
153	0.4854	a	0.0276	0.4851	a	0.0275	0.4853	a	0.0276
229.5	0.4726	a	0.0214	0.4732	a	0.0213	0.4727	a	0.0214
459	0.4935	a	0.0238	0.4936	a	0.0239	0.4931	a	0.0239

Mean is the average Pearson’s correlation and SE is the standard error. Different letters by the columns indicate statistical differences between periods with the Tukey test at 5% level of significance

#### Prediction accuracy under different regularization methods

Table 4 compares the prediction accuracy of the seven proposed methods using three regularization methods. For each method and number of basis, we compared three regularization methods –Bayes A, Bayes B and Bayes Lasso—using the ANOVA and Tukey procedures at the 5% level of significance. For each method and for each number of basis used, we did not find statistical differences between the three regularization methods in terms of prediction accuracy with Pearson’s correlation. This means that the three regularization methods are equally efficient in terms of predicting sample data in this particular data set. Table 4 also shows that comparing the three regularization methods in terms of implementation time for each method and number of basis, there were significant differences between the three regularization methods, where in general the most efficient was Bayes A and the most inefficient was Bayes Lasso; this was expected based on the nature of each regularization method.

**Table 4:** Prediction accuracy of grain yield with Pearson’s correlation for the 7 proposed methods, under BayesA, BayesB and Bayes Lasso (BL) for three numbers of basis (5, 29 and 51)

Method	Parameter	BayesA									BayesB									BayesLasso									Average	
		Number of basis									Number of basis									Number of basis										
		5			29			51			5			29			51			5			29			51				
		Pearson’s correlation									Pearson’s correlation									Pearson’s correlation										
M1	Mean	0.501	a	A	0.501	a	A	0.501	a	A	0.510	a	A	0.510	a	A	0.510	a	A	0.494	a	A	0.494	a	A	0.494	a	A	0.501	a
	SE	0.022			0.022			0.022			0.020			0.020			0.020			0.022			0.022			0.022			0.021	
M2	Mean	0.469	a	A	0.499	a	A	0.511	a	A	0.476	a	A	0.507	a	A	0.508	a	A	0.474	a	A	0.498	a	A	0.504	a	A	0.494	a
	SE	0.023			0.024			0.021			0.027			0.020			0.023			0.216			0.022			0.024			0.023	
M3	Mean	0.486	a	A	0.508	a	A	0.508	a	A	0.485	a	A	0.507	a	A	0.503	a	A	0.482	a	A	0.504	a	A	0.504	a	A	0.499	a
	SE	0.020			0.025			0.025			0.020			0.024			0.026			0.020			0.026			0.026			0.024	
M4	Mean	0.482	a	A	0.503	a	A	0.510	a	A	0.482	a	A	0.504	a	A	0.513	a	A	0.478	a	A	0.497	a	A	0.506	a	A	0.497	a
	SE	0.023			0.023			0.024			0.023			0.024			0.025			0.023			0.025			0.021			0.024	
M5	Mean	0.486	a	A	0.505	a	A	0.507	a	A	0.487	a	A	0.511	a	A	0.513	a	A	0.483	a	A	0.505	a	A	0.496	a	A	0.499	a
	SE	0.012			0.024			0.024			0.020			0.025			0.025			0.021			0.021			0.026			0.023	
M6	Mean	0.470	a	A	0.499	a	A	0.510	a	A	0.476	a	A	0.500	a	A	0.511	a	A	0.474	a	A	0.498	a	A	0.504	a	A	0.493	a
	SE	0.022			0.024			0.021			0.027			0.023			0.022			0.022			0.022			0.024			0.023	
M7	Mean	0.486	a	A	0.508	a	A	0.507	a	A	0.485	a	A	0.509	a	A	0.505	a	A	0.482	a	A	0.504	a	A	0.504	a	A	0.499	a
	SE	0.020			0.025			0.024			0.020			0.025			0.023			0.020			0.026			0.026			0.023	
		Implementation Time									Implementation Time									Implementation Time										
M1	Mean_T	30.911	a	A	30.911	a	A	30.911	a	A	30.911	a	A	30.911	a	A	30.911	a	A	30.911	a	A	30.911	a	A	30.911	a	A	30.911	a
	SE_T	2.16			2.16			2.16			2.16			2.16			2.16			2.16			2.16			2.16			2.16	
M2	Mean_T	9.180	c	C	11.233	c	C	11.106	de	A	10.170	c	B	12.160	c	B	11.093	c	A	12.720	c	A	15.340	b	A	9.900	c	A	11.430	c
	SE_T	0.08			0.05			0.08			0.02			0.2			0.54			0.03			0.08			0.22			1.02	
M3	Mean_T	9.230	c	B	10.826	c	C	10.910	e	A	9.893	c	B	12.176	c	B	10.973	c	A	12.190	c	A	15.113	bc	A	9.840	c	A	11.240	c
	SE_T	0.2			0.22			0.06			0.1			0.2			0.64			0.34			0.17			0.07			1	
M4	Mean_T	30.110	a	B	29.920	a	AB	27.186	b	A	30.110	a	B	26.746	b	B	26.310	b	AB	36.213	a	A	32.083	a	A	21.390	b	B	28.890	ab
	SE_T	1.08			1			0.05			0.9			0.03			0.94			1.46			0.78			1.73			2.46	
M5	Mean_T	26.720	b	B	26.426	b	B	26.633	c	A	26.620	b	B	26.463	b	B	26.030	b	A	32.390	b	A	31.776	a	A	21.463	b	B	27.170	b
	SE_T	0.08			0.14			0.18			0.13			0.2			0.6			0.3			0.8			1.62			1.87	
M6	Mean_T	8.060	c	C	9.423	c	C	11.353	d	A	8.676	c	B	10.433	d	B	11.980	c	A	10.806	c	A	13.360	bc	A	10.256	c	A	10.480	c
	SE_T	0.1			0.11			0.05			0.06			0.11			0.27			0.14			0.04			1.85			1.05	
M7	Mean_T	7.970	c	C	9.610	c	C	11.196	de	A	8.796	c	B	10.583	d	B	11.973	c	A	10.943	c	A	13.083	c	A	10.060	c	A	10.460	c
	SE_T	0.16			0.07			0.03			0.08			0.04			0.7			0.05			0.25			1.66			1.01	

Mean is the average Pearson’s correlation and SE is the standard error. Mean_T and SE_T are the average and standard error (in seconds) for implementing each scenario. The average column was calculated across the numbers of basis of the three methods by row. Different lowercase letters by the columns indicate statistical differences between methods with the Tukey test at 5% level of significance. Different uppercase letters by the rows indicate statistical differences between regularization methods with the Tukey test at 5% level of significance

## Discussion

Advances in computer power and in the technology for collecting and storing data considerably increased the presence of functional data whose graphical representations are curves, images or shapes. New types of data require new analytical tools, and functional data analysis is an area of statistics that extends conventional statistical methodologies and theories to the context of functional analysis. Generalized linear models, multivariate data analysis, nonparametric statistics and many other techniques are being expanded for the FRA framework. A key assumption in FRA and functional data analysis is that it is possible to approximate any curve onto a smaller space [23], with a series of basis functions by taking a weighted sum or linear combination of a sufficiently large number, *L*, of basis functions, as pointed out in Eq. (3).

One important assumption in functional data analysis is that it needs data that come from a smooth and continuous underlying process, understanding by smooth that the curve is differentiable to a certain degree; this implies that a number of derivatives can be obtained, although the observed data are subject to measurement error and other types of local disturbances that may mask this smoothness. Also, for truly functional data, there will be many more “covariables measured in time or any other continuum” than observations. However, virtually all data collection that we know comes from non-continuous observations, since samples are taken at discrete points in time or any other continuum. When we refer to discrete observations, the assumption is that there are enough observations to model the underlying process. It is important to recall that a typical functional data analysis begins by converting the raw data into functional objects. This is usually done using nonparametric smoothing techniques to represent each observation as a functional object. Then the original data are set aside, and the estimated curves themselves are used as input in subsequent analyses. This means that a two-stage process is used to analyze functional data and usually in the second stage it is possible to use conventional statistical methods. (However, this type of two-stage analysis is different from those implemented and proposed by other authors for analyzing multi-environment traits).

For the above reasons, FRA and functional data analysis application continues to increase in many areas of science because it offers a more complete framework for modeling the massive amounts of data that are collected and stored nowadays. Therefore, we view FRA as an important tool for building empirical models and for analyzing high-throughput phenotyping data in agriculture. Our application with real data highlights the value of background knowledge to be able to select the best FRA model and increase prediction accuracy. This means that successful FRA application depends strongly on many parameters that the practitioner needs to define as, for example, the type of basis functions (Fourier, B-splines, etc.), the required number of basis functions (*L*), the degree of the polynomial (*q*), the period (*T*), and the type of regularization method (BRR, BayesA, BayesB, Bayes Lasso), among others.

It is also very important to point out that one of the models proposed as an alternative for conventional regression analysis (given in Eq. (10), where only the design matrix, ***X***, was smooth) is very competitive with conventional functional regression (where both the design matrix and the beta coefficients are smooth) because it produces similar predictions, with the main advantage that it is more efficient computationally in implementation time and in parameter estimates (it produces more stable parameter estimates) because it has a lower dimension. Although not done here, in the linear predictor, the main effects of environments, genotypes, genotype × environment interaction terms and band × environment interaction terms can be taken into account; this usually helps to increase prediction accuracy, as reported by Montesinos-López et al. [17, 18]. It is also possible to incorporate genomic and/or pedigree information when available. Therefore, to those interested in understanding how to incorporate main effects, interaction terms and genomic and/or pedigree information, we suggest reading Montesinos-López et al. [17, 18] and the Additional file of this paper.

Based on our FRA application on a real data set and on our previous applications [17, 18], at least *L *= 23 was enough, but of course the particular data at hand should always be explored to determine the best choice of *L* to use. With regard to the degree of the polynomial in the context of B-splines, degree three is usually enough to approximate most of the curves quite well. The period is sometimes not easy to choose in the context of the Fourier basis, but in many applications the period is chosen as the difference between the maximum and minimum values of the time points measured, with satisfactory results in some cases.

With regard to the type of basis to use, the Fourier basis is frequently recommended for period curves and B-splines for non-periodic curves. However, when the number of basis is considerably large, both can be used for periodic or non-periodic curves. We feel that it is not a simple task to choose between the functional regression models implemented and proposed here. However, when the goal is prediction, two of the alternatives given here do a reasonable job (Alternative 2). The first one is the conventional regression model given in Eq. (8); the second one is the alternative 2 functional regression model given in Eq. (10), since its derivation is very intuitive and its corresponding predictor matrix of low dimension is more stable for estimating the required parameters. It also can reduce the computational time needed to implement it, when the value of *L* used is lower than the number of observations, *n*, to guarantee a well-defined regression problem.

In general, although traditional multiple regression methods can be used for analyzing functional data where an observation is a curve, they ignore the fact that the object underlying the measurements of a subject is a curve or a surface or any continuum. Zhang [31] pointed out that sometimes directly applying classic statistical methods is not straightforward for some of the following reasons: (a) the sampling time points of the observed functional data are not the same across various subjects; (b) the sampling time points are not equally spaced; and (c) the number of sampling time points is larger than the number of subjects in a sample of functional data. In the first scenario, direct classic statistical analysis may not be possible or reasonable; in the second scenario, classic statistical analysis inferences may be applied directly to the data, but whether the observed data really represent the underlying curves or surfaces may be questionable; in the third scenario, standard classic statistical analysis fails because the associated sample covariance matrix is degenerated so that most of the inference tools in classic statistical analysis will not be well defined. Many times dimension reduction techniques are applied first to solve these issues and often work well. However, in many situations, dimension reduction techniques may fail to reduce the dimension of the data sufficiently without losing too much information.

Therefore, in these situations, FRA is more natural and tends to have a higher signal-to-noise ratio in each observed value, because it allows extracting additional information contained in the functions and their derivatives, which is not normally possible using traditional methods. However, the modeling process is more complex than traditional statistical analysis, and in general the best tool for fitting FRA successfully to our data is an art combining knowledge of the data-generating process and the spirit of experimentation for testing various options.

## Conclusions

In this paper, we promote the use of functional regression modeling as an alternative for analyzing high-throughput phenotypic data and also provide the advantages and disadvantages of its implementation. In addition, many key elements that are needed to understand and implement this statistical technique appropriately are provided using a real data set. Of the two alternative models proposed in this paper, the second alternative given in Eq. (10), where only the design matrix (***X***) is smooth, is very attractive because: (a) it provides stable parameter estimates; (b) it considerably reduces implementation time; (c) its derivation is very intuitive; and (d) the prediction accuracy it provides is similar to that of the conventional model. Additionally, we provide a framework for implementing Bayesian functional regression using the GFR package developed based on the BGLR package, which nowadays is frequently used in the context of genomic selection. The advantage of using GFR for functional regression is that it is a powerful tool in the context of large *p* and small *n* problems that are mostly the rule in genomic data, since it allows using various regularization methods such as Bayesian Ridge regression, Bayes A, Bayes B, Bayes Lasso and Bayes C. For these reasons, we believe that this paper is a good guide for breeders and scientists who are interested in using functional regression models as tools for implementing prediction models when their data are curves.

### Additional file

**Additional file 1.** Installation of the GFR R package and additional examples.

## Appendix A: Deriving the two alternative proposed models

We can approximate each curve, $x_{i} \left( \varvec{t} \right)$, by replacing $\varvec{c}_{i}$ with $\hat{\varvec{c}}_{i} = \left[ {{\varvec{\Phi}}^{T} {\varvec{\Phi}}} \right]^{ - 1} {\varvec{\Phi}}^{T} x_{i} \left( \varvec{t} \right)$ in Eq. (3). Therefore, each row of the matrix design ***X*** was approximated by

11 $$\hat{x}_{i}^{T} \left( \varvec{t} \right) = x_{i}^{T} \left( \varvec{t} \right){\varvec{\Phi}}\left[ {{\varvec{\Phi}}^{T} {\varvec{\Phi}}} \right]^{ - 1} {\varvec{\Phi}}^{T}$$

By stacking the *n* rows with Eq. (11) for $i = 1, \ldots ,n$, we end up with an approximate design matrix, ***X***^*^, that is equal to

12 $$\varvec{X}^{*} = \left[ {\begin{array}{*{20}c} {x_{1}^{T} \left( \varvec{t} \right){\varvec{\Phi}}\left[ {{\varvec{\Phi}}^{T} {\varvec{\Phi}}} \right]^{ - 1} {\varvec{\Phi}}^{T} } \\ \vdots \\ {x_{n}^{T} \left( \varvec{t} \right){\varvec{\Phi}}\left[ {{\varvec{\Phi}}^{T} {\varvec{\Phi}}} \right]^{ - 1} {\varvec{\Phi}}^{T} } \\ \end{array} } \right] = \varvec{X}{\varvec{\Phi}}\left[ {{\varvec{\Phi}}^{T} {\varvec{\Phi}}} \right]^{ - 1} {\varvec{\Phi}}^{T}$$

It is important to point out that ***X*** and ***X***^*^ are both of order *n *× *m*, but of column rank *m* and L, respectively (where L is the number of basis). Then, as an approximation of the functional regression given in Eq. (1), we can regress the vector of response variable ***Y*** against the approximate design matrix of curves, ***X***^*^, resulting in the following traditional linear model:

13 $$\varvec{Y} = \varvec{X}^{*}\varvec{\beta}_{*} + \varvec{E}$$

with ***X***^*^ as given in Eq. (12); $\varvec{\beta}_{*}$ are beta coefficients of order *m *× 1 and ***E*** is a vector of errors as previously defined. Using the model given in Eq. (13), the corresponding least square estimate is given by $\hat{\varvec{\beta }}_{*} = \left( {\varvec{X}^{*T} \varvec{X}^{*} } \right)^{ - } \varvec{X}^{*T} \varvec{Y}$, and the predicted values are equal to: $\hat{\varvec{Y}} = \varvec{X}^{*} \hat{\varvec{\beta }}_{*} = \varvec{X}{\varvec{\Phi}}\left[ {{\varvec{\Phi}}^{T} {\varvec{\Phi}}} \right]^{ - 1} {\varvec{\Phi}}^{T} \left[ {{\varvec{\Phi}}\left[ {{\varvec{\Phi}}^{T} {\varvec{\Phi}}} \right]^{ - 1} {\varvec{\Phi}}^{T} \varvec{X}^{T} \varvec{X}{\varvec{\Phi}}\left[ {{\varvec{\Phi}}^{T} {\varvec{\Phi}}} \right]^{ - 1} {\varvec{\Phi}}^{T} } \right]^{ - 1} {\varvec{\Phi}}\left[ {{\varvec{\Phi}}^{T} {\varvec{\Phi}}} \right]^{ - 1} {\varvec{\Phi}}^{T} \varvec{X}^{T} \varvec{Y}$. Also, for prediction purposes, we can reparametrize the model given in Eq. (13) as:

14 $$\varvec{Y} = \varvec{X}^{*}\varvec{\beta}_{*} + \varvec{E} = \varvec{X}{\varvec{\Phi}}\left[ {{\varvec{\Phi}}^{T} {\varvec{\Phi}}} \right]^{ - 1} {\varvec{\Phi}}^{T}\varvec{\beta}_{*} = \varvec{X}^{**}\varvec{\beta}_{**} + E$$

where $$\varvec{X}^{**} = \varvec{X}{\varvec{\Phi}}$$ and $$\varvec{\beta}_{**} = \left[ {{\varvec{\Phi}}^{T} {\varvec{\Phi}}} \right]^{ - 1} {\varvec{\Phi}}^{T}\varvec{\beta}_{*}$$. Now ***X***^**^ is of order *n *× *L* and $$\varvec{\beta}_{**}$$ of order *L *× 1.
