# Correction to: Bayesian functional regression as an alternative statistical analysis of high-throughput phenotyping data of modern agriculture

**DOI:** 10.1186/s13007-018-0321-8

**Published:** 2018-07-09

**Authors:** Abelardo Montesinos-López, Osval A. Montesinos-López, Gustavo de los Campos, José Crossa, Juan Burgueño, Francisco Javier Luna-Vazquez

**Affiliations:** 1 Departamento de Matemáticas, Centro Universitario de Ciencias Exactas e Ingenierías (CUCEI), Universidad de Guadalajara, 44430 Guadalajara, Jalisco Mexico; 2 Facultad de Telemática, Universidad de Colima, 28040 Colima, Colima Mexico; 3 Epidemiology and Biostatistics and Statistics and Probability Departments, Michigan State University, 909 Fee Road, East Lansing, MI 48824 USA; 4 Biometrics and Statistics Unit, International Maize and Wheat Improvement Center (CIMMYT), Apdo. Postal 6-641, 06600 Mexico City, Mexico

## Correction to: Plant Methods (2018) 14:46 10.1186/s13007-018-0314-7

Unfortunately, in the original version [1] of this article, a funder note was missed out in the acknowledgement. The corrected acknowledgement is given below:

**Acknowledgements**

The authors thank all the field and lab assistants of CIMMYT’s Global Wheat Breeding Program who collected and processed the agronomic and breeding field data as well as the image data. The data used in this study was collected under projects supported by Bill and Melinda Gates Foundation and USAID.
