# Supplementary material for: Bayesian functional regression as an alternative statistical analysis of high-throughput phenotyping data of modern agriculture
Source: Plant Methods. 2018 Jun 11;14:46. doi: 10.1186/s13007-018-0314-7 (PMC5994840; doi:10.1186/s13007-018-0314-7)
Supplement: Supplementary file 1 — Additional file 1. Installation of the GFR R package and additional examples. [file 13007_2018_314_MOESM1_ESM.docx]

**Additional file 1**

**Bayesian functional regression as an alternative statistical analysis of high-throughput phenotyping data of modern agriculture**

**SUPPLEMENTARY MATERIAL-PART A**

**SA1. Installation of the GFR R package**

To install the genomic functional regression (GFR) package from GitHub, we need first install devtools package.

**install.packages**('devtools')

Using the install_github() function we can download and install the package,

devtools::**install_github**('frahik/GFR')

Now the package is ready to use.

In this paper, the version used was GFR 0.9-7

## SA.2. Examples

### **Example 1: Predictions with a data set with 100 lines, 3 environments with 250 bands, using Fourier basis.**

This example shows how to study prediction accuracies when the data have 100 lines, three environments, and 250 bands. This is done using the wheat_GFR dataset included in the package.

The following code block is used to load this dataset:

**library**(GFR)

**data**('Wheat_GFR')

With this, we load three objects named: (a) Wheat_GFR, a data.frame object which contains in tidy format the observations, the identifiers of the lines, the environments, (b) Wheat_Bands, a matrix with dimensions of 300 rows and 250 columns with the information of the bands, and (c) Wheat_Wavelenghts a vector with the names of the bands of length 250.

**head**(Wheat_GFR)

## Response Line Env

## 1 1.587324 3827768 Drought

## 2 3.140629 6176013 Drought

## 3 3.145934 4905617 Drought

## 4 0.984776 6931494 Drought

## 5 2.936291 6932344 Drought

## 6 1.882823 6935856 Drought

To fit a predictive model, we need three things, the first correspond to a list with the cross-validation type and number of folds (in the case of KFold cross-validation). Also, we need the linear predictor, this can be generated manually or using the ETAGenerate() function, in this case since the dataset Wheat_GFR contains environments, lines and bands we propose the following linear predictor:

$$\eta=Env+Line+Env\times Line+Bands$$

To specified this predictor inside the functions the following parameter are required: (a) basisType that corresponds to the type of basis to be implemented (Fourier or B-splines) (b) Bands is the matrix that contains all the bands, (c) Wavelengths is a vector that contains the names of each band measured, (d) priorType the Bayesian prior to be implemented by the model (e) method where you need to specified the method of estimation of the functional regression model (three types of methods are available, simplex (without band information), conventional (is the conventional FRA), Alternative1 and Alternative2, and finally (f) nBasis where you need to specified the number of basis.

After we generated the two objects (cross-validation list and ETA list) we are ready to fit a predictive model using the BFR() function with the **GFR** package, however given that the ETAGenerate() function contains in addition of the linear predictor also the data set which was used to generate the linear predictor for this reason it is not necessary to specify the data set in the function BFR. This mean that in the BFR function we only need to insert the linear predictor (object resulting of using the ETAGenerate()), the number of iterations (nIter) to adjust the Bayesian model, the numeric value for the burning (burnIn), the list with the folds for implementing the type of cross-validation selected (CrossValidation), and a seed (set_seed) for a replicable research. For example:

CrossV <- **list**(Type = 'KFold', nFolds= 3)

ETA1 <- **ETAGenerate**(Wheat_GFR, basisType = 'Fourier.Basis',

Bands = Wheat_Bands, Wavelengths = Wheat_Wavelengths, priorType = 'BayesA',

method= 'Alternative2', nBasis = 21)

PM1 <- **BFR**(ETA = ETA1, nIter = 15000, burnIn = 10000,

CrossValidation = CrossV, set_seed = 10)

When the model is ready, you can see the summary of the results with the summary() function, as follows

**summary**(PM1)

## Fold Env Trait Pearson SE_Pearson MSEP SE_MSEP Time

## 1 1 Irrigated 0.1301 NA 0.1402 NA 14.3600

## 2 1 Drought 0.3840 NA 0.3550 NA NA

## 3 1 ReducedIrrigated 0.2251 NA 0.1089 NA NA

## 4 2 ReducedIrrigated 0.3133 NA 0.1952 NA 12.6500

## 5 2 Drought 0.6626 NA 0.2359 NA NA

## 6 2 Irrigated 0.2805 NA 0.2991 NA NA

## 7 3 Drought 0.6573 NA 0.3344 NA 10.6900

## 8 3 ReducedIrrigated 0.1845 NA 0.1321 NA NA

## 9 3 Irrigated -0.1072 NA 0.5636 NA NA

## 10 Average_all Irrigated 0.1011 0.1128 0.3343 0.1235 12.5667

## 11 Average_all Drought 0.5679 0.0920 0.3084 0.0368 NA

## 12 Average_all ReducedIrrigated 0.2410 0.0380 0.1454 0.0258 NA

The summary provide information of the prediction accuracies (with Pearson´s correlation and mean square error of prediction (MSEP)) for each fold and for each environment under study (of course if the data has more than one environment). Additionally, provides the average of prediction accuracies under both metrics of the folds implemented for each environment. Also, this summary provide the time of implementation of each fold, and the NAs that appear is because each fold appear repeated depending of the number of environments. Also, it’s possibly get a plot the summary of the predictions with the Pearson´s correlation with its corresponding confidence interval using,

**plot**(PM1)


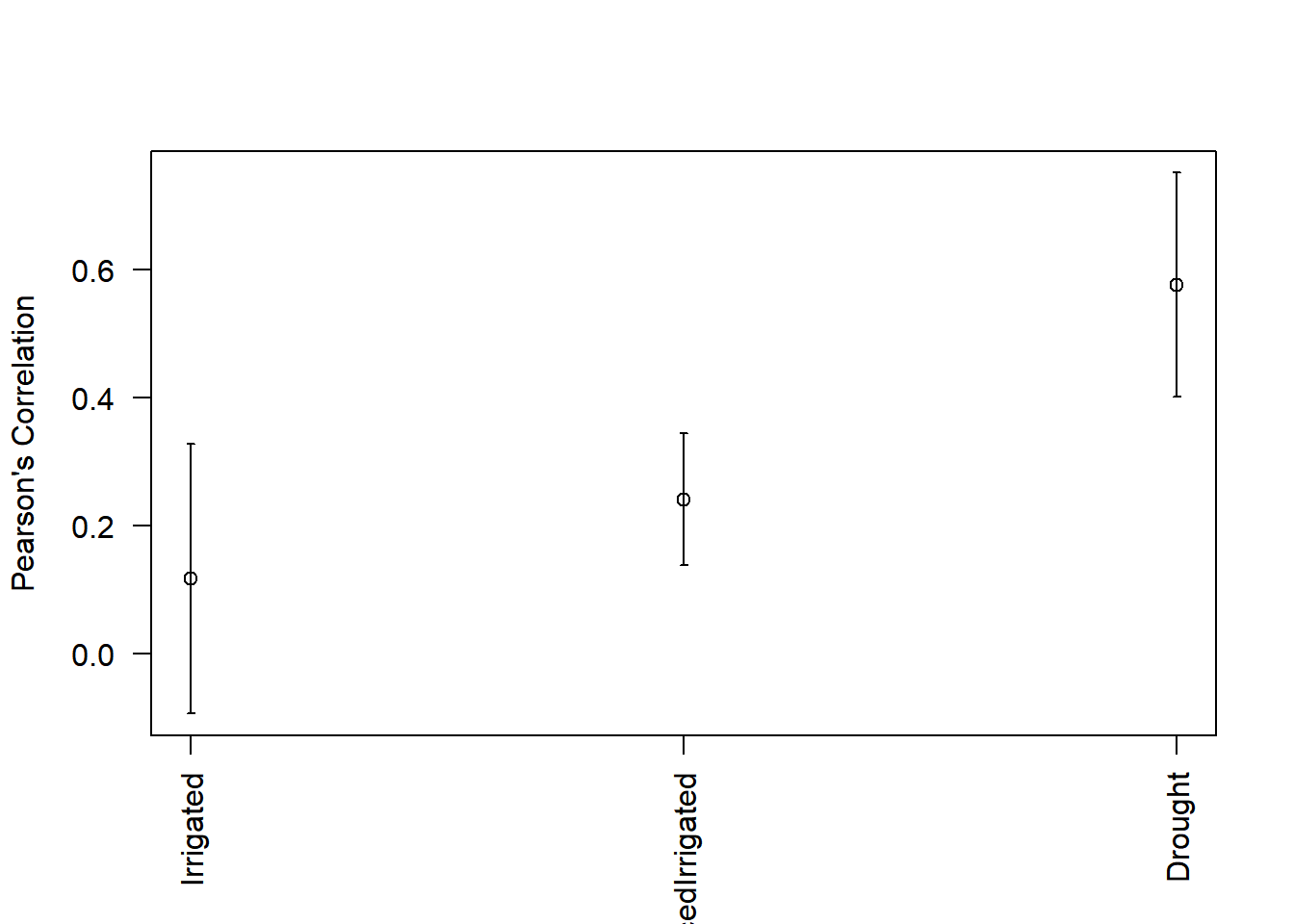


But, if we want the above results in terms of MSEP we can use the code: plot(PM1, select='MSEP'). Also, it’s possibly get a boxplot as summary of the prediction accuracy in terms of Pearson’s correlation, using the boxplot() function,

**boxplot**(PM1)


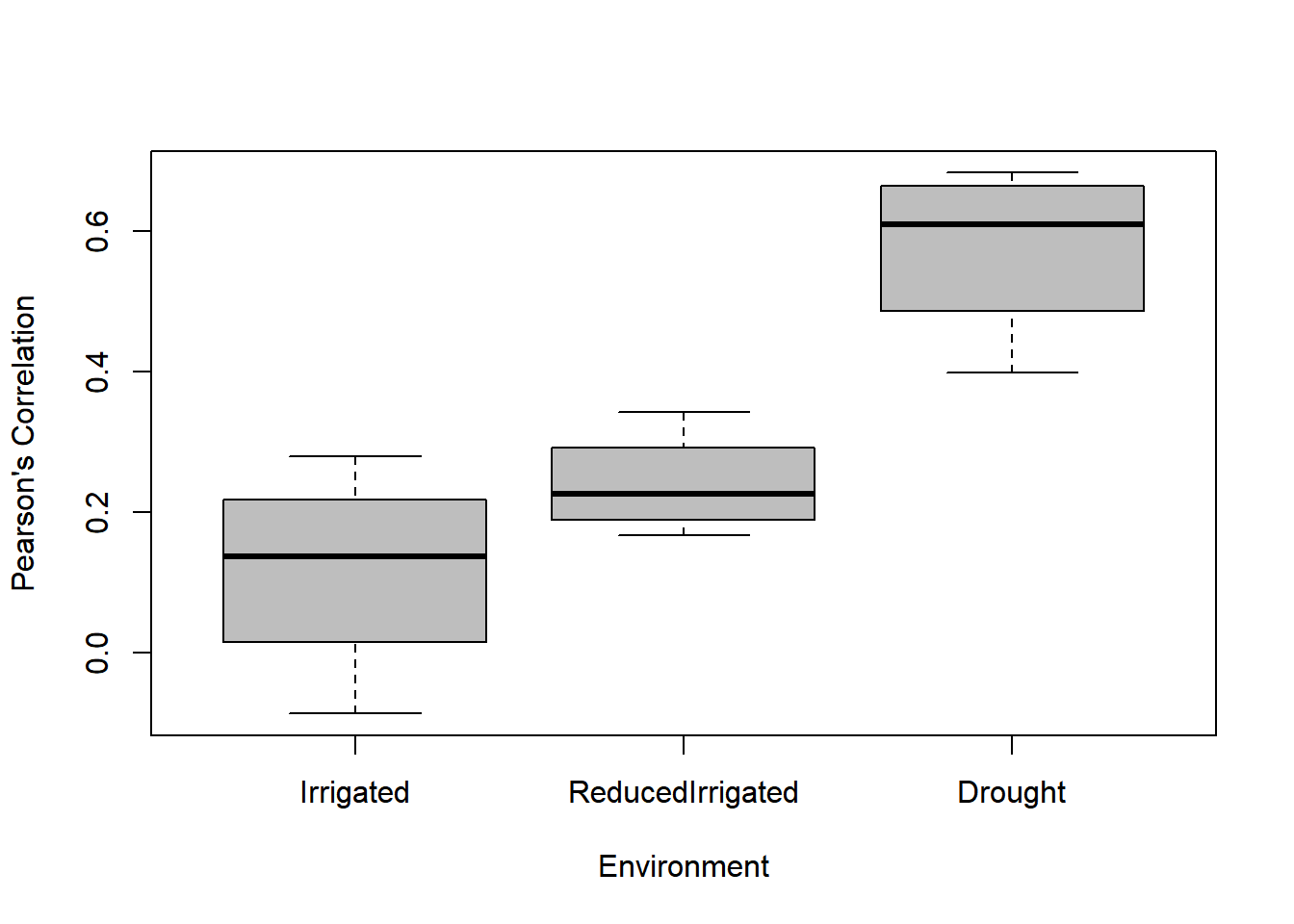


Also, we can get the boxplot in terms of MSEP with the code: boxplot(PM1, select='MSEP').

Next we illustrate how to use the GFR package for fitting the same data but without implementing a cross-validation. That is, we want to fit the predictor given above but to the whole data set. This is done with the following blocks of R code:

ETA2 <- **ETAGenerate**(Wheat_GFR, functionalType = 'Bspline.Basis', Bands = Wheat_Bands,

Wavelengths = Wheat_Wavelengths, priorType = 'BayesB',

method= 'Alternative2', nBasis = 21)

FM2 <- **BFR**(ETA = ETA2, nIter = 15000, burnIn = 10000)

This implementation only differ from the last one that now we not created the list for the cross-validation and when it is used the BFR () function not used the CrossValidation argument.

To see the information that we can extract from this fitted model to the whole data set we can use the str() function.

**str**(FM1)

## List of 21

## $ response : num [1:300] 1.587 3.141 3.146 0.985 2.936 ...

## .

## .

## .

## $ predictions : num [1:300] 2.15 2.5 2.48 1.27 2.37 ...

## $ SD.predictions: num [1:300] 0.216 0.255 0.238 0.217 0.209 ...

## $ mu : num 3.67

## $ SD.mu : num 0.0741

## $ varE : num 0.248

## $ SD.varE : num 0.027

## $ fit :List of 4

## ..$ logLikAtPostMean: num -167

## ..$ postMeanLogLik : num -193

## ..$ pD : num 50.8

## ..$ DIC : num 436

## $ ETA :List of 4

## ..$ Env :List of 24

## .. ..$ model : chr "BayesB"

## .. ..$ Name : chr "ETA_Env"

## .

## .

## .

## ..$ Line :List of 24

## .. ..$ model : chr "BayesB"

## .. ..$ Name : chr "ETA_Line"

## .

## .

## .

## ..$ LinexEnv:List of 24

## .. ..$ model : chr "BayesB"

## .. ..$ Name : chr "ETA_LinexEnv"

## .

## .

## .

## ..$ Bands :List of 24

## .. ..$ model : chr "BayesB"

## .. ..$ Name : chr "ETA_Bands"

## .

## .

## .

## - attr(*, "class")= chr "BFR"

### **Example 2. Predictions for a dataset with 3 environments, 100 lines and Bands with a handmade linear estimator.**

This example shows how to study prediction accuracies when the data have three environments, 100 lines and bands and we need a specific predictor (ETA) for the model. Again we will use the wheat_GFR dataset included in the package.

If we only want to study the main effects of environments, lines and bands the following predictor can be of interest:

$$\eta=Env+Line+Bands$$

With different priors for each component of the predictor it is not possible to use the ETAGenerate() function explained above, however next we shown how to create at hand a predictor where each main effect has its particular prior distribution:

ETA3 <- **list**(Env = **list**(X =**model.matrix**(~0+**as.factor**(Wheat_GFR$Env)), model = 'BRR'),

Line = **list**(X = **model.matrix**(~0+**as.factor**(Wheat_GFR$Line)), model = 'BRR'),

Bands = **list**(X = **data.matrix**(Wheat_Bands), model = 'BayesA'))

After this we need to create the cross-validation specification and we are ready to fit the predictive model,

CrossV <- **list**(Type = 'KFold', nFolds=3)

PM2 <- **BFR**(data = Wheat_GFR, ETA = ETA3, nIter = 15000, burnIn = 10000,

CrossValidation = CrossV, set_seed = 10)

A summary of the prediction accuracy can be obtained with the summary() function and if we want to plot the MSEP for each environment under study we can use the following code:

**plot**(PM2, select = 'MSEP')


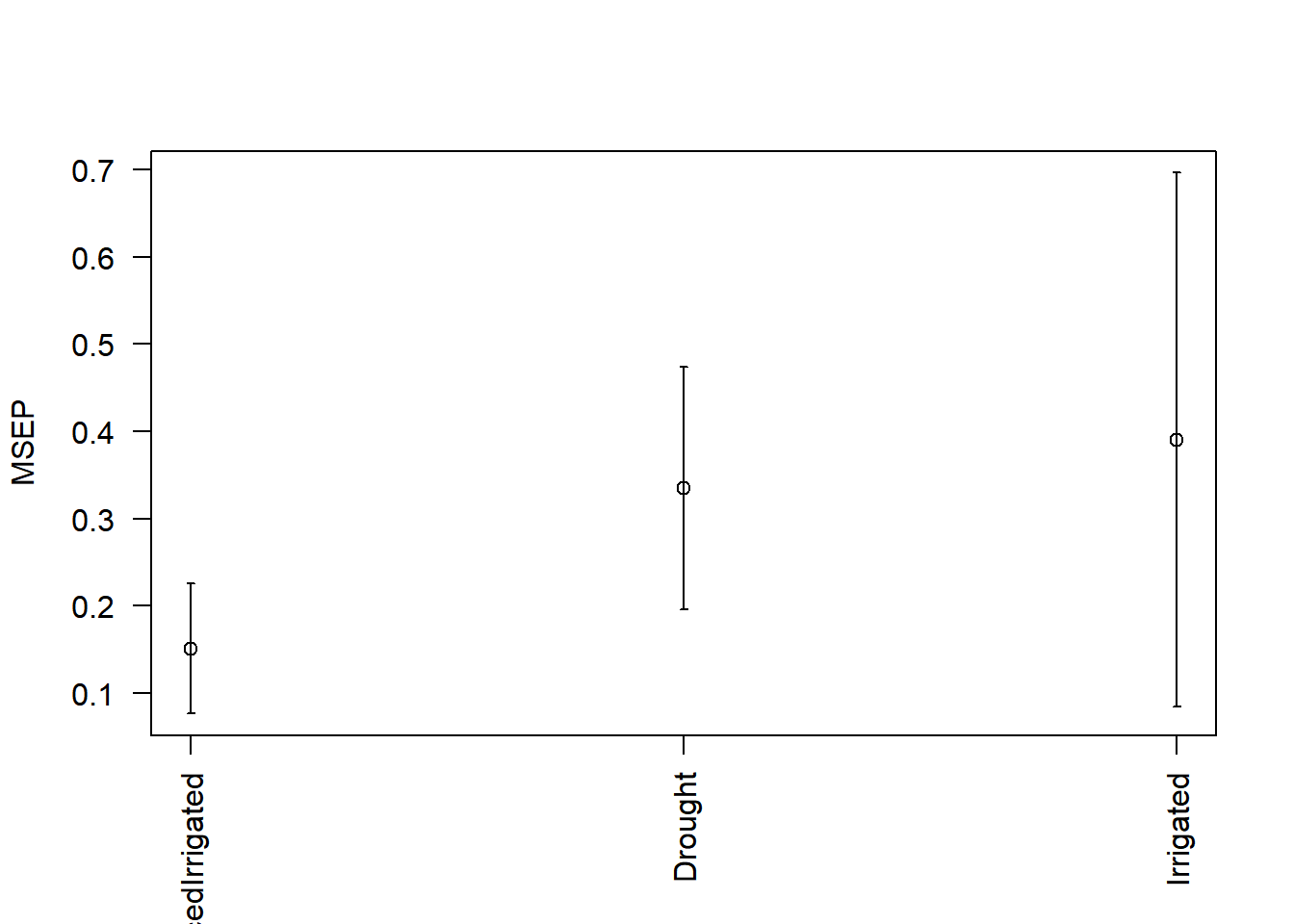


On the other hand, if you want to see a boxplot as summary of the prediction accuracies for each environment the next code is the correct one:

**boxplot**(PM2, select = 'MSEP')


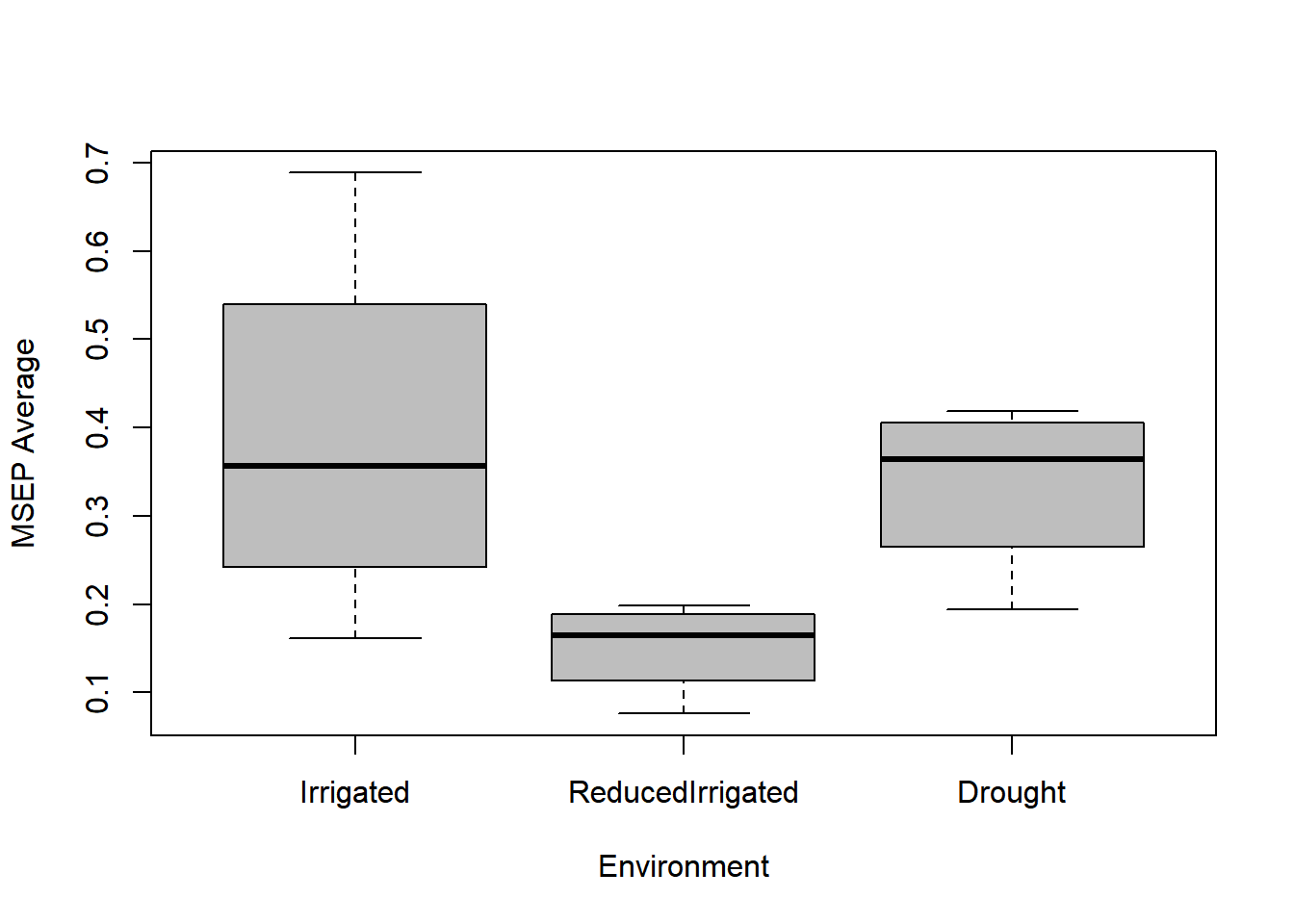


**Example 3. Fitting a prediction model with a single environment, a single trait and many bands using Fourier Basis with the prior BayesC**

This example shows how to fit a model when the data set only contains one trait, one environment and many bands using Fourier basis with prior BayesC.

To fit the model, we need filter the data set and the bands, in the next code we show how to filter the dataset Maize_GFR also available in the GFR package. With this filtering process we only get information for Environment ‘KAK’ and Trait ‘PH’.

**data**('Maize_GFR')

dataset <- Maize_GFR[**which**(Maize_GFR$Env == 'EBU'),]

dataset <- dataset[**which**(dataset$Trait == 'Yield'), ]

bands <- Maize_Bands[1:309, ]

wavelengths <- Maize_Wavelengths

If we only want to study the main effects of the bands the following predictor can be of interest:

$$\eta=Bands$$

Like we already filter the dataset with one environment and one trait, the function ETAGenerate() produces this predictor automatically, to do this we can use the following code,

ETA4 <- **ETAGenerate**(dataset, basisType = 'Fourier.Basis', Bands = bands,

Wavelengths = wavelengths, priorType = 'BayesC', method = 'Alternative2',

nBasis = 21)

After this we need the list with the type of cross-validation that we will implement, in this case we will use a random partition cross-validation, with the next code we are ready to fit the predictive model,

CrossV <- **list**(Type = 'RandomPartition', NPartitions = 5, PTesting = 0.3)

It is important that with Type = 'RandomPartition' we are ordering to the GFR package to create a list of NPartitions = 5 random partitions, where in each of this random partitions 30% of data are assigned to the testing set and the remaining 70% of the data are assigned to the training data set.

Using the main function BFR() we can fit this model,

PM4 <- **BFR**(ETA = ETA4, data, nIter = 15000, burnIn = 10000, set_seed = 10,

CrossValidation = CrossV)

A summary of the prediction accuracy can be obtained with the summary() function with the following code,

**summary**(PM4)

## Fold Env Trait Pearson SE_Pearson MSEP SE_MSEP Time

## 1 1 EBU Yield -0.0950 NA 0.6492 NA 2.290

## 2 2 EBU Yield -0.0886 NA 0.9214 NA 2.240

## 3 3 EBU Yield 0.0017 NA 0.8313 NA 1.290

## 4 4 EBU Yield -0.1726 NA 0.8852 NA 1.440

## 5 5 EBU Yield 0.0059 NA 0.7306 NA 1.370

## 6 Average_all EBU Yield -0.0697 0.0335 0.8036 0.0502 1.726

**Example 4. Fitting a Multi-trait and Multi-environment model with Fourier basis and with the BayesC prior**

In this case, we show how to fit a model when we have 3 environments, 3 traits and many bands. To load the Maize_GFR dataset we must use the following code,

**data**('Maize_GFR')

Assume that you want to implement the following linear predictor

$$\eta=Env+Trait+Line+Line\times Env+Line\times Trait+Env\times Trait+Env\times Trait\times Line+Bands\times Env$$

The implementation of this predictor using the **ETAGenerate**() is not straight forward since this function only cannot be implemented by default. When the data are multi-trait and multi-environment without bands the following predictor can be implemented by default in the GFR package:

$$\eta=Env+Trait+Line+Line\times Env+Line\times Trait+Env\times Trait+Env\times Trait\times Line$$

And when the data are multi-trait and multi-environment with bands, the following predictor also can be implemented by default:

$$\eta=Env+Trait+Line+Line\times Env+Line\times Trait+Env\times Trait+Env\times Trait\times Line+Bands$$

So, to be able to implement the first linear predictor in the context of multi-trait and multi-environment. First we generate the linear predictor-for multi-trait and multi-environment without bands- with the following code,

ETA5 <- **ETAGenerate**(Maize_GFR, priorType = 'BayesC')

And to add the $Env\times Band$ term in the linear predictor, we add to the ETA5 generated above by hand, the $Env\times Band$ term as follows,

ETA5$ETA$EnvxBands <- **list**(X = **Fourier.Basis**(Maize_Bands, Maize_Wavelengths,

nBasis = 21, interaction = Maize_GFR$Env), model = 'BayesC')

Since the linear predictor is ready, next we provide the R code to create the cross-validation, and to fit the predictive model,

CrossV <- **list**(Type = 'RandomPartition', NPartitions = 5, PTesting = 0.3)

PM5 <- **BFR**(ETA = ETA5, data, nIter = 15000, burnIn = 10000, set_seed = 10,

CrossValidation = CrossV)

A summary of the prediction accuracy is obtained with the summary() function with the following code,

**summary**(PM5)

## Fold Env Trait Pearson SE_Pearson MSEP SE_MSEP Time

## 1 1 EBU ASI 0.0076 NA 0.7741 NA 319.22

## 2 1 KAK ASI -0.0481 NA 2.0212 NA NA

## 3 1 KTI ASI -0.0616 NA 2.1805 NA NA

## 4 1 EBU PH 0.0384 NA 140.7002 NA NA

## 5 1 KAK PH 0.1520 NA 167.9120 NA NA

## 6 1 KTI PH 0.1618 NA 227.4074 NA NA

## 7 1 EBU Yield -0.0739 NA 1.0606 NA NA

## 8 1 KAK Yield -0.0582 NA 1.1353 NA NA

## 9 1 KTI Yield 0.0286 NA 1.8174 NA NA

## 10 2 EBU ASI 0.0389 NA 3.4295 NA 312.43

## 11 2 KAK ASI 0.1007 NA 2.0597 NA NA

## 12 2 KTI ASI -0.1155 NA 5.7314 NA NA

## 13 2 EBU PH 0.0679 NA 399.8994 NA NA

## 14 2 KAK PH 0.1361 NA 127.1688 NA NA

## 15 2 KTI PH 0.0416 NA 247.9218 NA NA

## 16 2 EBU Yield -0.0261 NA 4.1928 NA NA

## 17 2 KAK Yield 0.0542 NA 1.6363 NA NA

## 18 2 KTI Yield -0.1354 NA 6.0393 NA NA

## 19 3 EBU ASI -0.0889 NA 3.5002 NA 306.82

## 20 3 KAK ASI -0.0156 NA 2.3799 NA NA

## 21 3 KTI ASI -0.1793 NA 1.5885 NA NA

## 22 3 EBU PH 0.1269 NA 150.9429 NA NA

## 23 3 KAK PH 0.2543 NA 119.5058 NA NA

## 24 3 KTI PH 0.1148 NA 201.6865 NA NA

## 25 3 EBU Yield 0.0192 NA 3.5527 NA NA

## 26 3 KAK Yield 0.0069 NA 1.3770 NA NA

## 27 3 KTI Yield 0.0724 NA 1.5598 NA NA

## 28 4 EBU ASI -0.1540 NA 0.9108 NA 311.11

## 29 4 KAK ASI 0.0622 NA 2.3479 NA NA

## 30 4 KTI ASI 0.1227 NA 1.1004 NA NA

## 31 4 EBU PH 0.0414 NA 385.3245 NA NA

## 32 4 KAK PH -0.0221 NA 142.6006 NA NA

## 33 4 KTI PH 0.2211 NA 203.2408 NA NA

## 34 4 EBU Yield 0.0172 NA 1.2528 NA NA

## 35 4 KAK Yield -0.1359 NA 2.0373 NA NA

## 36 4 KTI Yield 0.0116 NA 1.2856 NA NA

## 37 5 EBU ASI 0.0163 NA 32.7293 NA 306.92

## 38 5 KAK ASI -0.1096 NA 1.4626 NA NA

## 39 5 KTI ASI -0.0047 NA 13.9060 NA NA

## 40 5 EBU PH -0.0030 NA 159.0312 NA NA

## 41 5 KAK PH 0.2117 NA 186.0570 NA NA

## 42 5 KTI PH 0.0326 NA 260.0483 NA NA

## 43 5 EBU Yield 0.1155 NA 31.3265 NA NA

## 44 5 KAK Yield 0.0264 NA 1.1738 NA NA

## 45 5 KTI Yield 0.0558 NA 13.5553 NA NA

## 46 Average_all EBU ASI -0.0360 0.0367 8.2688 6.1432 311.30

## 47 Average_all KAK ASI -0.0021 0.0378 2.0543 0.1648 NA

## 48 Average_all KTI ASI -0.0476 0.0515 4.9014 2.3937 NA

## 49 Average_all EBU PH 0.0543 0.0214 247.1796 59.4882 NA

## 50 Average_all KAK PH 0.1464 0.0471 148.6488 12.4854 NA

## 51 Average_all KTI PH 0.1144 0.0358 228.0609 11.6827 NA

## 52 Average_all EBU Yield 0.0104 0.0313 8.2771 5.7952 NA

## 53 Average_all KAK Yield -0.0213 0.0341 1.4719 0.1670 NA

## 54 Average_all KTI Yield 0.0066 0.0370 4.8515 2.3444 NA

It is important to point out that since the time of implementation was obtained for each fold for this reason only in one of the trait-environment combinations is different of NAs and this values represent the time of implementation of each fold. Also you can use plot(PM5) to get the plot with the predictions accuracies for each trait-environment combination with the Pearson´s correlation or use plot (PM5, select = 'MSEP') to get the predictions for the trait-environment combination in terms of MSEP. Also, you can get the boxplot of the summary of the prediciton accuracies with boxplot (PM5) or with boxplot (PM5, select='MSEP') in terms of Pearson´s correlation or MSEP respectivelly.

**SUPPLEMENTARY MATERIAL-PART B**

**SB1. Methods for knots location and knots number selection**

**Uniform knot-spacing method**. This method takes $K$ equally spaced points in the range of interest, say, [*a, b*], as interior knots. That is, *K* interior knots are defined as

$$\tau_{r}=a+\left( b-a \right)r/ (K+1), r =1, 2, \ldots, K.$$

This uniform knot-placing method is independent of the design time points and is usually employed when the design time points are believed to be uniformly scattered in the range of interest (Zhang, 2013). For example, if the range of interest is between $a=10$ and $b=20$, and assuming that $K=5$, then: $\tau_{1}=10+\frac{\left( 20-10 \right)1}{\left( 5+1 \right)}= 10+\frac{10}{6}=11.66$, $\tau_{2}=10+\frac{\left( 20-10 \right)2}{\left( 5+1 \right)}= 10+\frac{20}{6}=13.33$, $\tau_{3}=10+\frac{\left( 20-10 \right)3}{\left( 5+1 \right)}= 10+\frac{30}{6}=15$, $\tau_{4}=10+\frac{\left( 20-10 \right)4}{\left( 5+1 \right)}= 10+\frac{40}{6}=16.66$, $\tau_{5}=10+\frac{\left( 20-10 \right)4}{\left( 5+1 \right)}= 10+\frac{50}{6}=18.33$.

**Quantiles as knots method.** This method uses equally spaced simple quantiles of the design time points $t_{i,}i=1, 2, \ldots, m$ as knots. Let $t_{(1)}, \ldots, t_{(m)}$ be the order statistics of the design time points. Then the *K* interior knots are defined as $\tau_{r}= t_{(1+[rn/(K+1)]),} r=1, 2, \ldots, K,$ where [a] denotes the integer part of a. This quantiles as knots method is design adaptive. It locates more interior knots where more design time points are available.

When the design time points are uniformly scattered, this is approximately equivalent to the uniform interior knot spacing method (Zhang, 2013). Also, assuming that the range of interest is between $a=10$ and $b=20$, *m*=100 time points and assuming that $K=5$, $t_{(1)}, \ldots, t_{(100)}$, then $\tau_{1}= t_{\left( 1+\left[ \frac{1\times100}{5+1} \right] \right)}=t_{\left( 17 \right)}$, $\tau_{2}= t_{\left( 1+\left[ \frac{2\times100}{5+1} \right] \right)}=t_{\left( 34 \right)}$, $\tau_{3}= t_{\left( 1+\left[ \frac{3\times100}{5+1} \right] \right)}=t_{\left( 51 \right)}$, $\tau_{4}= t_{\left( 1+\left[ \frac{4\times100}{5+1} \right] \right)}=t_{\left( 67 \right)}$, $\tau_{5}= t_{\left( 1+\left[ \frac{5\times100}{5+1} \right] \right)}=t_{\left( 84 \right)}$.

After specifying a knot-placing method, the number of interior knots, *K*, can be chosen by the following generalized cross-validation (GCV) rule:

$$GCV\left( K \right)= \frac{||y-\hat{y}_{L}||^{2}}{(1-tr(\boldsymbol{H}_{L})/n)^{2}}$$

where $L=K+q+1, tr \left( \boldsymbol{H}_{L} \right)=L$, and $y$ and $\hat{y}_{L}$are the response vector and the fitted response vector, respectively. The regression spline smoother matrix $\boldsymbol{H}_{L}$ is an idempotent matrix, satisfying that the transpose of matrix $\boldsymbol{H}_{L}$ is equal to $\boldsymbol{H}_{L}$and that $H_{L}^{2}$= $\boldsymbol{H}_{L}$ and ${tr\boldsymbol{(}\boldsymbol{H}}_{L})=L$. The trace of the smoother matrix $\boldsymbol{H}_{L}$ is often called the degrees of freedom of the regression spline smoother. It measures the complexity of the fitted regression spline model [18]. Also, we can use it as a criterion to select the number of interior knots, K, the coefficient of determination, $R^{2}$, defined as follows:

$R^{2}\left( K \right)= cor(y,\hat{y}_{L})^{2}$,

where$cor$ denotes the Pearson correlation (or Spearman correlation), and the required number of interior knots, K, can be selected defining a minimum threshold for $R^{2}$. For example, we can select K as the minimum value that guarantees a value of $R^{2}$ of at least 0.98. Of course, that depending of the threshold defined a priori (in terms of $R^{2} )$ should be the required number of interior knots, K, and the closer the value of $R^{2}$ to 1, the larger the required number of interior knots, K.

The goal of these two criteria for selecting K and, indirectly, $L$, is to make certain that $L$ is large enough to fit the data well and considerably reduce the dimension of the original data so as to be able to considerably reduce the computation time.

**SB2. Assumptions on prior distributions of the seven proposed models**

For Bayesian implementation of the proposed methods in Table 1, we assume that the variance component error term, $\sigma_{e}^{2},$ was a scaled inverse Chi-square distribution $\chi^{-2}(\sigma_{e}^{2}|S_{e}, df_{e}$), with scale value $S_{e}=0.1$ and degrees of freedom $df_{e}=5$. For the beta coefficients of methods M1, M2, M3, M4, M5, M6 and M7 under a Bayesian Ridge regression (BRR) approach for $\beta_{k}, k=1,\ldots,p \mathrm{or} L,$ we assumed an $N\left( 0\boldsymbol{,}\sigma_{\beta_{1}}^{2} \right)$, where $N\left( a\boldsymbol{,}b \right)$ stands for a normal distribution with mean $a$ and variance$b$, and for $\sigma_{\beta_{1}}^{2}\boldsymbol{\sim}\chi^{-2}(\sigma_{\beta_{1}}^{2}|S_{\beta_{1}}, df_{\beta_{1}}$), while under BayesA, the marginal distribution of each beta coefficient has a scaled-t density, with parameters $df_{\beta_{k}}$ and $S_{\beta_{k}}$. This density is implemented as an infinite mixture of scaled-normal densities; at the first level of the hierarchy, beta coefficients are assigned normal densities with mean zero and specific variance $\sigma_{\beta_{k}}^{2}$, $k=1,\ldots,p \mathrm{or} L$. At the second level of the hierarchy, these variance parameters are independently assigned and identically distributed (iid) scaled-inverse Chi-squared densities with degree of freedom and scale parameters$df_{\beta_{k}}$ and $S_{\beta_{k}}$, respectively (de los Campos and Pérez-Rodriguez, 2014).

For the BayesB model, the beta coefficients are assigned iid priors that are mixtures of a point of mass at zero and a slab that is a scaled-t density. The slab is structured as in BayesA. Therefore, BayesB extends BayesA by introducing an additional parameter *π* which in the case of GFR represents the prior proportion of non-zero effects. This parameter is treated as unknown and assigned a Beta prior *π ∼ Beta*($p_{0}$*;* $\pi_{0}$), with $p_{0}$*>* 0 and $\pi_{0}\epsilon$[0*;* 1]. The beta prior is parameterized in such a way that the expected value by *E*(*π*) = $\pi_{0}$; the variance of the Beta distribution is then given by *V ar*(*π*) = *π*0(1*- π*0) (de los Campos and Pérez-Rodriguez, 2014).

Under the Bayesian LASSO (BL) model, the marginal distribution of beta coefficients is double exponential. At the first level of the hierarchy, the beta coefficients are assigned independent normal densities with null mean and beta coefficient-specific variance parameter $\tau_{jk}^{2}\times\sigma_{e}^{2}$. The residual variance is assigned a scaled-inverse Chi-square density, and the beta coefficient-specific scale parameters, $\tau_{jk}^{2}$, are assigned iid exponential densities with rate parameter$\lambda^{2}/2$. Finally, at the last level of the hierarchy, $\lambda^{2}$ was assigned a Gamma distribution with shape and scale parameters $r$ and $s$ (de los Campos and Pérez-Rodriguez, 2014).

**SB3**. Proof that $\boldsymbol{\Phi}^{T}\boldsymbol{\Phi}\approx\frac{1}{\Delta}\int_{0}^{1} \phi_{i}\left( t \right)\phi_{j}\left( t \right)dt$

Note that by the definition of the Riemann integral, we can express

$$\int_{0}^{1} \phi_{i}\left( t \right)\phi_{j}\left( t \right)dt\approx\sum_{k=1}^{m} \Delta_{k}\phi_{i}\left( t_{k} \right)\phi_{j}\left( t_{k} \right)$$

$$\approx\sum_{k=1}^{m} \Delta\phi_{i}\left( t_{k} \right)\phi_{j}\left( t_{k} \right); \Delta_{k}= \Delta$$

$$\Rightarrow\sum_{k=1}^{m} \phi_{i}\left( t_{k} \right)\phi_{j}(t_{k})\approx\frac{1}{\Delta} \int_{0}^{1} \phi_{i}\left( t \right)\phi_{j}\left( t \right)dt$$

Therefore, if $\boldsymbol{\Phi}^{T}\boldsymbol{\Phi}=\left\{ P_{ij} \right\}; i=1,2,\ldots,L$ and $j=1,2,\ldots,S$

where $P_{ij}= \sum_{k=1}^{m} \phi_{i}\left( t_{k} \right)\phi_{j}(t_{k})$

$$\Rightarrow P_{ij}\approx\frac{1}{\Delta} \int_{0}^{1} \phi_{i}\left( t \right)\phi_{j}\left( t \right)dt=\frac{1}{\Delta}J_{ij}$$

where $J_{ij}= \int_{0}^{1} \phi_{i}\left( t \right)\phi_{j}\left( t \right)dt$; therefore:

$$\boldsymbol{\Phi}^{T}\boldsymbol{\Phi}\approx\frac{1}{\Delta}J; \text{with} J=\{J_{ij}\}$$

This implies that $\hat{\boldsymbol{Y}}$ resulting from conventional regression using equation (8) should be

$$\hat{\boldsymbol{Y}}=\boldsymbol{W}\left( \boldsymbol{W}^{T}\boldsymbol{W} \right)^{-1}\boldsymbol{W}^{T}\boldsymbol{Y}$$

where $\boldsymbol{W}=\boldsymbol{X}\boldsymbol{\Phi}\left[ \boldsymbol{\Phi}^{\boldsymbol{T}}\boldsymbol{\Phi} \right]^{\boldsymbol{-1}}\boldsymbol{J};\boldsymbol{J}\approx\Delta\boldsymbol{\Phi}^{T}\boldsymbol{\Phi}$

$$\boldsymbol{W}=\boldsymbol{X}\boldsymbol{\Phi}\left[ \boldsymbol{\Phi}^{T}\boldsymbol{\Phi} \right]^{-1}\Delta\boldsymbol{\Phi}^{T}\boldsymbol{\Phi}$$

$$=\boldsymbol{X}\boldsymbol{\Phi}\Delta$$

This implies that

$$\hat{\boldsymbol{Y}}=\left[ \boldsymbol{X}\boldsymbol{\Phi}\Delta\right]\left[ \Delta\boldsymbol{\Phi}^{T}\boldsymbol{X}^{T}\boldsymbol{X}\boldsymbol{\Phi}\Delta\right]^{-1}\boldsymbol{X}\boldsymbol{\Phi}\Delta\boldsymbol{Y}$$

$$=\Delta^{2}\boldsymbol{X}\boldsymbol{\Phi}\left[ \boldsymbol{\Phi}^{T}\boldsymbol{X}^{T}\boldsymbol{X}\boldsymbol{\Phi} \right]^{-1}\Delta^{-2}\boldsymbol{X}\boldsymbol{\Phi}\boldsymbol{Y}$$

$$=\boldsymbol{X}\boldsymbol{\Phi}\left[ \boldsymbol{\Phi}^{T}\boldsymbol{X}^{T}\boldsymbol{X}\boldsymbol{\Phi} \right]^{-1}\boldsymbol{X}^{T}\boldsymbol{\Phi}\boldsymbol{Y}$$
